# Modeling the cumulative genetic risk for multiple sclerosis from genome-wide association data

**DOI:** 10.1186/gm217

**Published:** 2011-01-18

**Authors:** Joanne H Wang, Derek Pappas, Philip L De Jager, Daniel Pelletier, Paul IW de Bakker, Ludwig Kappos, Chris H Polman, Lori B Chibnik, David A Hafler, Paul M Matthews, Stephen L Hauser, Sergio E Baranzini, Jorge R Oksenberg

**Affiliations:** 1 Department of Neurology, University of California San Francisco, San Francisco, CA 94143-0435, USA; 2 Program in Translational NeuroPsychiatric Genomics, Department of Neurology, Brigham and Women's Hospital and Harvard Medical School, Boston, MA 02115, USA; 3 Program in Medical and Population Genetics, Broad Institute of Harvard University and Massachusetts Institute of Technology, Cambridge, MA 02139, USA; 4 Division of Genetics, Department of Medicine, Brigham and Women's Hospital and Harvard Medical School, Boston, MA 02115, USA; 5 Department of Neurology, University Hospital Basel, CH 4031, Basel, Switzerland; 6 Department of Neurology, Vrije Universiteit Medical Centre, Amsterdam 1007 MB, The Netherlands; 7 Florey Neuroscience Institutes, University of Melbourne, Victoria 3053, Australia; 8 Department of Neurology, Yale University, New Haven, CT 06520-8018, USA; 9 GlaxoSmithKline Clinical Imaging Centre, Hammersmith Hospital and Department of Clinical Neurosciences, Imperial College, London W12 0NN, UK; 10 Institute for Human Genetics, School of Medicine, University of California San Francisco, San Francisco, CA 94143-0435, USA

## Abstract

**Background:**

Multiple sclerosis (MS) is the most common cause of chronic neurologic disability beginning in early to middle adult life. Results from recent genome-wide association studies (GWAS) have substantially lengthened the list of disease loci and provide convincing evidence supporting a multifactorial and polygenic model of inheritance. Nevertheless, the knowledge of MS genetics remains incomplete, with many risk alleles still to be revealed.

**Methods:**

We used a discovery GWAS dataset (8,844 samples, 2,124 cases and 6,720 controls) and a multi-step logistic regression protocol to identify novel genetic associations. The emerging genetic profile included 350 independent markers and was used to calculate and estimate the cumulative genetic risk in an independent validation dataset (3,606 samples). Analysis of covariance (ANCOVA) was implemented to compare clinical characteristics of individuals with various degrees of genetic risk. Gene ontology and pathway enrichment analysis was done using the DAVID functional annotation tool, the GO Tree Machine, and the Pathway-Express profiling tool.

**Results:**

In the discovery dataset, the median cumulative genetic risk (P-Hat) was 0.903 and 0.007 in the case and control groups, respectively, together with 79.9% classification sensitivity and 95.8% specificity. The identified profile shows a significant enrichment of genes involved in the immune response, cell adhesion, cell communication/signaling, nervous system development, and neuronal signaling, including ionotropic glutamate receptors, which have been implicated in the pathological mechanism driving neurodegeneration. In the validation dataset, the median cumulative genetic risk was 0.59 and 0.32 in the case and control groups, respectively, with classification sensitivity 62.3% and specificity 75.9%. No differences in disease progression or T2-lesion volumes were observed among four levels of predicted genetic risk groups (high, medium, low, misclassified). On the other hand, a significant difference (F = 2.75, *P *= 0.04) was detected for age of disease onset between the affected misclassified as controls (mean = 36 years) and the other three groups (high, 33.5 years; medium, 33.4 years; low, 33.1 years).

**Conclusions:**

The results are consistent with the polygenic model of inheritance. The cumulative genetic risk established using currently available genome-wide association data provides important insights into disease heterogeneity and completeness of current knowledge in MS genetics.

## Background

Multiple sclerosis (MS) is a common cause of non-traumatic neurological disability in young adults. Extensive epidemiological and laboratory data indicate that genetic susceptibility is an important determinant of MS risk [1,2]; this risk is modulated by family history, ancestry, gender, age, and geography [3]. The extent of familial clustering is often expressed in terms of the λ_s _parameter derived from the ratio between the risk seen in the siblings of an affected individual and the risk seen in the population [4]. In northern Europeans, the prevalence is 1 per 1,000 in the population and the recurrence risk in a sibling is 2 to 3%; hence, after correcting for age, the λ_s _for MS is approximately 15 to 20. On the other hand, some authors suggest that both of these risks are difficult to assess and the denominator is generally underestimated while the numerator is overestimated [5,6]; a more accurate value for λ_s _may be less than 10 [7]. In addition, twin studies from several populations consistently show that a monozygotic twin of an MS patient is at higher risk for MS than is a dizygotic twin [8,9]; however, they vary in their estimation of indices of heritability from 0.25 to 0.76 [10].

MS behaves as a prototypic complex genetic disorder, and although a single-gene etiology cannot be ruled out for a subset of pedigrees, data from recent genome-wide association studies (GWAS) provide convincing evidence that support a multifactorial and polygenic model of inheritance [11-14]. It is also likely that epistatic and epigenetic events modulate heritability [15-18]. The human leukocyte antigen (*HLA*) gene cluster in chromosome 6p21.3 represents by far the strongest MS susceptibility locus genome-wide. The primary signal maps to the *HLA-DRB1 *gene in the class II segment of the locus, but complex hierarchical allelic and/or haplotypic effects and protective signals in the class I region between *HLA-A *and *HLA-C *have been reported as well [2,19-21]. Other susceptibility genes discovered primarily through GWAS include *IL2RA*, *IL7R*, *EVI5*, *CD58*, *CLEC16A*, *CD226*, *GPC5*, and *TYK2 *[11,12,14,22-25]. A recent meta-analysis of data from three different GWAS totaling 2,624 MS patients and 7,220 controls identified additional susceptibility SNPs within or next to *TNFRSF1A*, *ICSBP1*/*IRF8 *and *CD6 *[24]. In addition to gene discovery, these studies are powering a profound paradigm shift in the study of MS by allowing a more accurate description of the genetic contributions to disease susceptibility [26]. Even though the full roster of MS genes remains unknown at this time, we build on the meta-analysis dataset and use logistic regression methodology to estimate the collective genetic risk behind MS susceptibility. In line with other complex diseases [27], the results remain consistent with the polygenic paradigm and suggest that while much of the genetics of MS remains to be characterized, up to 350 independent variants account for a significant fraction of the genetic component of MS.

## Materials and methods

### Data

A genome-wide meta-analysis of MS was recently completed and reported [24]. Since each of the three pooled studies used a different genotyping platform, we use data from the phased chromosomes of HapMap samples of European ancestry [28] and the MACH algorithm [29] to impute missing autosomal SNPs with a minor allele frequency >0.01 in each of the datasets. Fractional genotypic scores are generated as the outcome of MACH imputation algorithm, and are analyzed without converting them into categorical genotypes to minimize variance inflation. The distribution of fractional genotype scores are tri-modal with the peaks at 0, 1 and 2, but there are data points that fall in between peaks due to uncertainty encountered during the imputation process. The estimated variance inflation factor was λ = 1.077. The final discovery dataset included 8,844 samples (2,124 cases and 6,720 controls) and a common panel of 2.56 million SNPs (Table 1). The independent validation dataset is composed of 1,618 ANZgene cases and 1,988 controls [12]. We used MACH to impute the ANZgene dataset as described for the discovery dataset.

**Table 1:** Demographic statistics of study participants

	Discovery dataset (N = 8,844)		Validation dataset^b ^(N = 3,606)	
		
	Case	Control	Case	Control
Stratum^a^	(N = 2,124)	(N = 6,720)	(N = 1,618)	(N = 1,988)
IMSGC UK, Affy 500K	17.5%	40.9%	-	-
IMSGC US, Affy 500K	13.2%	23.3%	-	-
BWH, Affy 6.0	32.2%	23.9%	-	-
Gene MSA CH, Illumina 550K	9.6%	2.9%	-	-
Gene MSA NL, Illumina 550K	8.9%	3.1%	-	-
Gene MSA US, Illumina 550K	18.6%	5.9%	-	-
Male	27.9%	50.3%	27.5%	38.1%
Female	72.1%	49.7%	72.5%	61.9%
DRB1*15:01 +	52.7%	25.1%	56.9%	29.8%
DRB1*15:01 -	47.3%	74.9%	43.1%	70.2%

^a^Datasets described in [24]. In each pair of matched cases and controls, all subjects are genotyped using the same genome-wide platform. ^b^Datasets described in [12], with 1,618 Australian and New Zealand cases (Illumina Hap370CNV) matched with 1,988 US controls (Illumina Infinium).

### Statistical analysis

All statistical analyses were performed using SAS v.9.1.3 and JMP Genomics v. 4.0 (SAS Institute, Cary, NC 27513, USA). Principle component analysis was implemented prior to data analysis to assess population substructure. Although no significant population substructure was observed when compared to the HapMap CEU data, a few outliers were removed. We organize the top association analysis results (*P *< 0.001) of the meta-analysis in the discovery dataset by individual chromosomes and implement a logistic regression analysis using alternation between the type I and type III sums of squares tests to remove markers that are in linkage disequilibrium (LD). The top ranked SNPs (that is, the SNP with the most extreme *P*-value) are forced into the model first. We then calculate the residual effect of each of the other SNPs after accounting for the effect of the top ranked SNPs. We used gender and sample country of origin (US versus EU, total 6 stratum) as covariates in the model to account for possible population heterogeneity. Furthermore, conditional logistic regression was implemented conditioning on DRB1*15:01 status (Yes versus No) in order to control the effect of genetic heterogeneity. This method is preferred to the conventional logistic regression model in estimating the gene risk effect after 'conditioning out' the baseline risk in DRB1*15:01 carriers and non-carriers, and it is thus efficient in eliminating the redundancy of markers that are in LD with DRB1*15:01. HLA-DRB1*15:01 status was determined using a tagging marker (rs3135388).

Logistic regression stepwise selection was applied to select a set of genes from the identified independent markers and establish a genetic profile to assess the cumulative genetic risk of individuals (P-Hat). Logistic regression is used for prediction of the probability of occurrence of an event by fitting data to a logit function. It is a generalized linear model used for binomial regression. The logit of the unknown binomial probabilities (P-Hat) is modeled as a linear function of the *Xi*, with a set of explanatory variables, where logit (P-Hat) = ln(P-Hat/1 - P-Hat) = *β_0_+β_1_X_1_+β_2_X_2_+···+BiXi*; and thus, P-Hat = 1/1+ exp^-(*β0 + β1X1 + β2X2 + ···+BiXi*)^. The algorithm for calculating the predicted probability is modeled after an event being a MS case, P-Hat = 1/(1+ exp(-Ŷi)), where Ŷi = intercept + *βcenter *× *Xcenter *+ *βgender *× *Xgender *+ ∑*βj*×*Xij*; *βj *is the estimated regression coefficient of genetic marker *j*, and *j *= 1 to 350; *Xij *is the fractional genotype of marker *j *of individual *i*. The values of intercept, *βcenter*, *βgender*, and *βj *are the maximum likelihood estimates obtained from the logistic regression model. The regression coefficient reflects the differential contribution of each SNP, and the odds ratio is estimated by exponentiating the corresponding regression coefficient. In order to assess how well the genetic profile can differentiate MS cases from the controls, the cumulative genetic risk classification is performed. If Ŷi of an individual is >0, then the individual is classified as a MS case, and if Ŷi is <0, then they are classified as a control. When Ŷi = 0, the estimated probability of being an MS patient is 0.5.

Classification sensitivity and specificity are assessed. Classification sensitivity is defined as the percentage of affected individuals that are classified as an MS case, and specificity as the percentage of controls that are classified as a control. Analysis of covariance (ANCOVA) was implemented to compare clinical characteristics of individuals with various degrees of genetic risk (high, medium, low and misclassified group), with gender as covariate in the model. The Hosmer-Lemeshow goodness-of-fit test was implemented to test if the observed probability is equal to the expected probability based on the fitted model; a *P*-value <0.05 indicates a lack of fit of the fitted logistic regression model [30].

### Functional gene ontology and annotation

Gene ontology enrichment analysis was done using the DAVID functional annotation tool [31] and GO Tree Machine, and pathway enrichment was done with the Pathway-Express profiling tool [32], using default parameters and correcting for multiple comparison by the Benjamini method and the false discovery rate (FDR), respectively.

## Results

The characteristics of the discovery (8,844 samples) and validation datasets (3,606 samples) are shown in Table 1. The frequency of HLA-DRB1*15:01 was similar across the disease groups. As expected, gender ratios were different between cases and controls in all datasets. Gender was fit into the model for all subsequent analyses to minimize the effect of this difference. Using the top 12 validated disease variants for MS including *HLA-DRB1 *(Additional file 1), we estimated the collective genetic risk in the discovery dataset, yielding a classification sensitivity of 35.1% and a specificity of 93.5% (Table 2), suggesting the presence of many additional susceptibility alleles in the strata of data that failed to achieve genome-wide significance. We then tested whether a significant fraction of the variance was related to contributions from additional common alleles with lower association effects. The analysis was conducted in four major stages: stage I, genome-wide association analysis; stage II, LD filtering; stage III, statistical model fitting using the independent markers identified in stage II; and stage IV, validation in an independent replication dataset.

**Table 2:** Estimated cumulative genetic risk using 12 validated multiple sclerosis genes^a^

	Probability of being a MS case		
	
	25% quartile	Median	75% quartile
Case (N = 2,062)	0.228	0.379	0.589
Control (N = 6,360)	0.072	0.134	0.268

Classification results in the discovery dataset were: classification sensitivity, 35.1%; classification specificity, 93.5%; classification accuracy rate, 63.8%; model fit analysis, *P *= 0.007 (Hosmer-Lemeshow goodness-of-fit test [30] was implemented to assess 'lack of fit' of the selected model; *P *> 0.05 indicates that there is no evidence of a lack of fit of the selected model). ^a^*HLA-DRB1*, *CD58*, *CLEC16a*, *EVI5*, *IL2Ra*, *IRF8*, *RGS1*, *CD226*, *TNFRSF1a*, *CD6*, *GPC5 *and *IL7R*.

### Stage I analysis

Case-control logistic regression analysis was implemented on the discovery dataset with 8,844 samples (2,124 cases versus 6,720 controls). Two regression models were applied. The first model included center and gender as covariates, whereas the second model included center, gender and DRB1*15:01 status as covariates. A relatively lax threshold of significance was chosen to compensate for the lack of statistical power to detect minor effects. Markers with *P*-value <0.001 (equivalent to controlling FDR at 25%) from both analyses were selected for further study. Altogether, 11,334 markers (0.44% of the 2.56 million markers) were included in the stage II analysis.

### Stage II analysis

The main objective of the stage II analysis was to trim redundancy among the 11,334 markers identified in the stage I analysis. Conditional logistic regression was used to remove markers in LD with DRB1*15:01 [33]. Covariates such as center and gender were placed in the model throughout the analysis. Two procedures were implemented; the first examined residual effects after preceding markers were placed in the model (type I sums of squares test, also known as sequential sums of squares test). The significant *P*-value from the type I test indicated that the marker showed an independent effect in addition to the preceding markers that were already placed in the model. The second test sought to examine multicollinearity in between markers due to LD (type III sums of squares test). The *P*-value from the type III test indicated if the marker of interest remained significant after all other markers were placed in the model. Thus, if any two markers in the model were in LD, one or both of the marker's *P*-value would not be significant. Markers that did not reach *P *< 0.01 from both type I and type III tests were removed. The flow chart of analysis procedures is shown in Additional file 2. We first selected the top significant markers at *P *< 10^-5 ^(the most significant markers per gene), then placed this set of markers into a logistic regression analysis in the sequence of significance to examine independence of markers (type I test). This first set of independent markers was then placed in a logistic regression model (type III test) to search for markers with remaining effect at *P *< 0.001. The second set of markers was then selected and combined with the first set of markers, and was examined using both type I and type III analyses in a logistic regression model again to examine independent effect and multicollinearity. Markers that did not show additional independent effects were removed. This expanded set of independent markers was then placed into a regression model (type III test) to search for additional independent markers. These steps were repeated until all markers with an independent effect at *P *< 0.001 were identified. The analysis identified 713 independent markers across all autosomal chromosomes, and included the original GWAS and meta-analysis associated markers (*CD58*, *CLEC16a*, *EVI5*, *IL2Ra*, *IRF8*, *RGS1*, *CD226*, *TNFRSF1a*, *CD6 *and *IL7R*). Markers with significance at -Log10 (p) > 6.0 are shown in Table 3. Markers exceeding significance at FDR = 0.05 are shown in Additional file 3.

**Table 3:** Top significant markers (-Log 10(p) > 6)) after adjusting for DRB1*15:0 1 among the 700-independent-gene set

rs ID	Position	Chrom.	Gene name	Allele 1	Allele 2	-Log10 p	OR	Lower CL	Upper CL
rs9268148	32367505	6	*C6orf10*	A	G	13.13	0.58	0.50	0.67
rs1611715	29937461	6	*HLA-G*	C	A	11.49	0.74	0.68	0.81
rs7772297	31436805	6	*HLA-B*	C	G	9.14	1.40	1.26	1.56
rs4939490	60550227	11	*CD6*	G	C	9.00	1.30	1.19	1.42
rs9275596	32789609	6	*HLA-DQA2*	T	C	7.85	0.76	0.69	0.84
rs10244467	22584456	7	*IL6*	T	C	7.23	0.57	0.47	0.70
rs9596270	49740441	13	*DLEU1*	T	C	7.08	1.56	1.31	1.85
rs12025416	116750329	1	*CD58*	C	T	6.83	0.69	0.59	0.80
rs6836440	100405684	4	*ADH4*	A	G	6.74	0.68	0.58	0.79
rs7137953	119357405	12	*GATC*	C	T	6.47	0.77	0.70	0.85
rs10846336	16413619	12	*MGST1*	T	C	6.43	0.42	0.30	0.59
rs931555	35839334	5	*IL7R*	C	T	6.41	1.25	1.15	1.36
rs10203141	179015804	2	*OSBPL6*	C	G	6.40	0.81	0.75	0.88
rs2328523	20575342	6	*E2F3*	G	A	6.28	0.79	0.72	0.87
rs4368946	98497864	8	*TSPYL5*	T	C	6.25	0.70	0.61	0.80
rs3934035	281714	3	*CHL1*	C	T	6.23	0.46	0.34	0.62
rs17062281	73654880	13	*KLF12*	C	G	6.13	0.44	0.31	0.61
rs1356122	155666264	3	*GPR149*	G	C	6.13	1.26	1.14	1.40
rs4447	31599694	22	*SYN3*	T	C	6.10	0.74	0.66	0.83
rs655763	108682027	11	*C11orf87*	C	T	6.03	1.59	1.32	1.92
rs12419184	125561518	11	*RPUSD4*	C	T	6.03	0.72	0.63	0.82

Chrom., chromosome; lower CL, lower bound of the confidence interval; OR, odds ratio; upper CL, upper bound of the confidence interval.

### Stage III analysis

Using the identified 713 independent markers, we performed a model fitting analysis to select the optimal set of variants that gave the best estimation of the cumulative genetic risk mediated by common alleles for an individual and that differentiated MS cases from controls. Logistic regression analysis using stepwise-selection with different selection entrance and remaining cutoff values (*P *= 0.01, *P *= 0.05, *P *= 0.1) was implemented. The stepwise-selection process included an alternation between forward selection of a set of significant markers and backward elimination of markers that did not retain significance at the selected threshold after additional markers were placed in the model. The stepwise selection process terminated when additional significant markers could not be fitted into the model. The covariates included in the logistic regression analysis were center and gender. This analysis identified 350 genes using *P *= 0.05 as the cutoff selecting criteria, including *CD58*, *EVI5*, *IRF8*, *RGS1*, *CD226*, *TNFRSF1a*, *CD6*, and *IL7R*. However, *IL2Ra*, *CLEC16a*, *IRF8*, and *HLA-C *did not survive the stepwise regression analysis.

**Figure 1:**
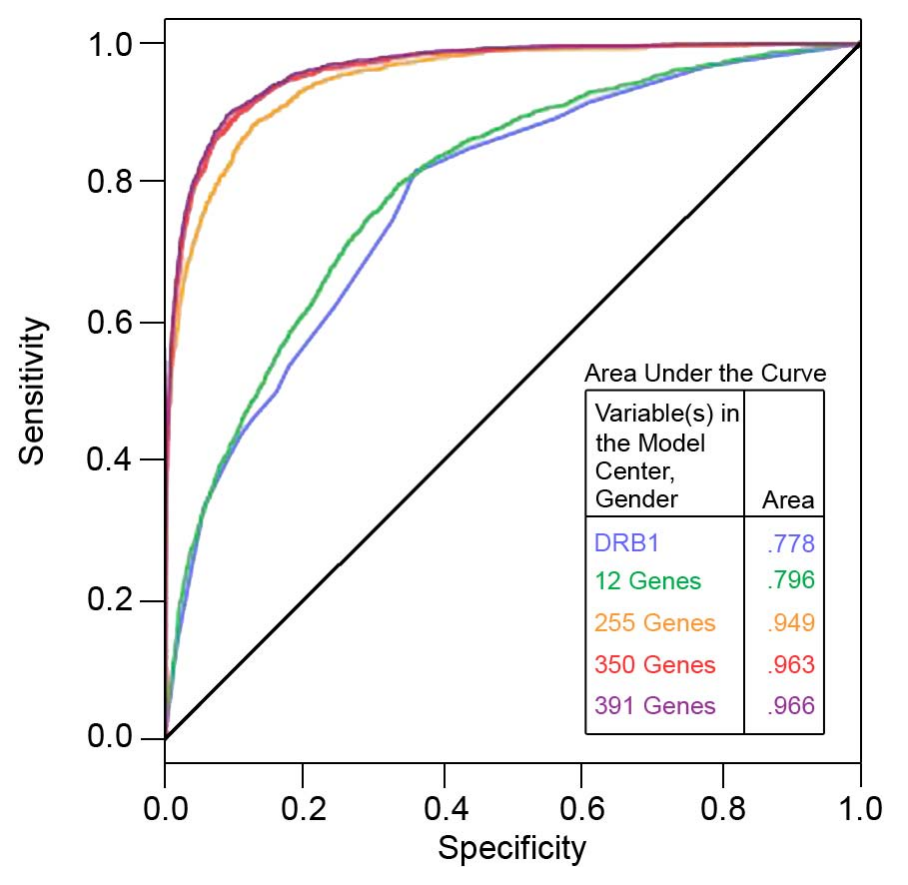
**ROC curves of different genetic models using the discovery dataset (N = 8,844)**. Stepwise selection from the 700-gene list yielded gene sets with different numbers of genes used in the predictive model: 255 genes (*P *= 0.01), 350 genes (*P *= 0.05), and 391 genes (*P *= 0.10).

The cumulative genetic risk for each individual was calculated using the estimated regression coefficients of the 350 markers included in the model, providing a measure of the extent to which common allelic variation (and the variables in the model) explained disease status in this dataset. The explanatory potential of these variables can be expressed as a summary estimate of the predicted probability of an individual being a MS case (P-Hat). The median of the cumulative genetic risk in the case group is 0.90, and in the control group 0.01. Quantiles of the estimated cumulative genetic risk (P-Hat) using different genetic models is summarized in Table 4. Next, classification sensitivity and specificity were assessed. In addition, receiver operating characteristic (ROC) analysis comparing classification results using different genetic models is shown in Figure 1. The classification results did not improve substantially when more markers were included using less stringent selection criteria (*P *= 0.10, 391 markers). Classification sensitivity only increased from 79.9% to 80.3%, and the adjusted R^2 ^only improved from 0.75 (*P *= 0.05, 350 markers) to 0.76 (391 markers). Therefore, we tested the predictive power of the selected 350 variants (Additional file 4). The Hosmer-Lemeshow goodness-of-fit test resulted in a *P*-value of 0.092, indicating that there is no evidence of a lack of fit or over-fitting in the selected model. As expected, this model has much better discriminating power than the 12-gene-set model (Table 4).

**Table 4:** Classification results using different genetic models

	Classification	Classification	P-Hat (quantiles, case versus control)		
			
Genetic model	sensitivity	specificity	25%	50%	75%
Discovery dataset (N = 8,844)					
12 Genes^a^	35.1%	93.5%	0.23 0.07	0.38 0.13	0.59 0.27
350 Genes^b^	79.9%	95.8%	0.65 0.00	0.90 0.01	0.99 0.06
					
Validation dataset (N = 3,606)					
12 Genes^a^	54.3%	74.0%	0.36 0.30	0.53 0.36	0.63 0.51
350 Genes^b^	62.3%	75.9%	0.41 0.19	0.59 0.32	0.74 0.49

^a^The 12-gene set includes *HLA-DRB1 *and 11 additional validated susceptibility genes. ^b^The 350-gene set includes *HLA-DRB1 *and 349 additional genes identified in the genetic profile.

### Stage IV analysis

**Figure 2:**
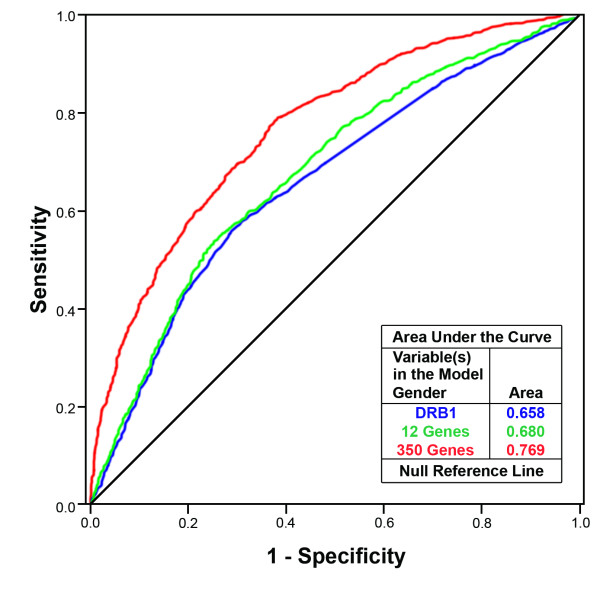
**ROC curves of different genetic models using the validation dataset (N = 3,606)**. Logistic regression using forward selection method. The 350 genetic markers were entered into the model by rank of significance.

The genetic profile established in the stage III analysis was tested on an independent dataset including 1,618 MS cases and 1,988 controls [12]. We used the same 350 genetic markers as predictors in a logistic regression model to calculate the predicted probability of being an MS patient, the median of the cumulative predicted genetic risk (P-Hat) in the case group is 0.59 and 0.32 in the control group. Quantiles of the estimated cumulative genetic risk (P-Hat) are given in Table 4. We then used the probability to classify individuals into cases or controls (if P-Hat of an individual is >0.5, then the individual is classified as a MS case, otherwise, a control). The classification results were used to assess sensitivity and specificity for the 3,606 independent samples; the statistics are shown in Table 4. The classification sensitivity is approximately 62.3%, which shows a moderate improvement compared to using the 12 validated genes (54.3%). The classification sensitivity is modest, reflecting the limited power of the study, randomness, heterogeneity, possible epistasis, and lack of fitting environmental and epigenetic factors into the model. We also performed a ROC analysis (ROC curve) in the validation dataset to compare the area under curves (AUCs) of various genetic models (Figure 2).

### Clinical characteristics of individuals with various degrees of genetic load

In order to further understand the significance of the affected individuals' cumulative genetic risk, patients with available clinical data in the screening dataset (N = 968) were grouped into four clusters using their predicted probability of being a MS patient (P-Hat): high (P-Hat ≥0.95, N = 383); medium (P-Hat <0.95 and ≥0.75, N = 313); low (P-Hat <0.75 and ≥0.5, N = 142); misclassified (P-Hat <0.5, N = 130). Not surprisingly, Chi-square testing for the association of genetic load with DRB1*15:01 status showed the strong effect of the allele or haplotype (high P-Hat: 63.2% in DRB1*15:01+ versus 36.8% in DRB1*15:01-), along with the decrease in the proportion of DRB1*15:01 carriers from the highest P-Hat group to the lowest P-Hat group: (high, 63.2%; medium, 46.6%; low, 35.9%; misclassified, 23.9%; *P *< 0.0001). Similar association was observed with gender (female) (high, 74.4%; medium, 65.8%; low, 59.9%; misclassified, 52.3%; *P *< 0.0001) (Table 5).

**Table 5:** Clinical and demographic characteristics of various genetic-load groups

	Genetic-load groups by the level of estimated cumulative genetic risk				
	
	High	Medium	Low	Misclassified	
Clinical and demographic variables	P-Hat ≥ 0.95	P-Hat = 0.75-0.95	P-Hat = 0.5-0.75	P-Hat < 0.5	Test
Sample size, N (%)	383 (39.6%)	313 (32.3%)	142 (14.7%)	130 (13.4%)	
MSSS (least-square mean)^a^	1.77	1.82	1.83	1.81	F = 0.41, *P *= 0.75^c^
T2-lesion load (mm^3^) (least-square mean)^b^	15.41	15.40	14.32	15.81	F = 0.98, *P *= 0.40^c^
Age of disease onset (years)	33.81	33.55	33.18	35.90	F = 2.71, *P *= 0.03^d^
DRB1*15:01 +, N (%)	242 (63.2%)	146 (46.7%)	51 (35.9%)	31 (23.9%)	χ^2 ^= 74.13^e^
DRB1*15:01 -, N (%)	141 (36.8%)	167 (53.4%)	91 (64.1%)	99 (76.1%)	*P *< 0.0001
Female, N (%)	285 (74.4%)	206 (65.8%)	85 (59.9%)	68 (52.3%)	χ^2 ^= 25.41^e^
Male, N (%)	98 (25.6%)	107 (34.2%)	57 (40.1%)	62 (47.7%)	*P *< 0.0001

^a^MSSS (Multiple Sclerosis Severity Score [44]) after square-root transformation to meet normality assumption. ^b^T2-lesion volumes after cube-root transformation to meet normality assumption. ^c^ANCOVA test result, with 'age of disease onset' and gender as covariates. ^d^ANCOVA test result, with gender as covariate.

^e^Chi-square test result.

Multiple Sclerosis Severity Score (MSSS), T2-lesion volumes (mm^3^), and age of disease onset (years) were analyzed using ANCOVA tests, with gender as covariate in the model. MSSS was transformed using square-root transformation for normality assumption. T2-lesion volumes (mm^3^) were transformed using cube-root transformation for normality assumption. The global test results did not show statistically significant difference between the four groups on MSSS (F = 0.41, *P *= 0.75) and T2-lesion volumes (F = 0.98, *P *= 0.40), whether age of disease onset was placed in the model as a covariate or not (MSSS, F = 0.41, *P *= 0.74; T2-lesion volumes, F = 0.69, *P *= 0.56). However, there was a significant difference in age of disease onset between the MS affected misclassified as controls (mean = 36 years) and the other three groups (high group, mean = 33.77 years; medium group, mean = 33.57 years; low group, mean = 33.23 years) (Table 5). Sib concordance in multi-case family studies show that age of onset is the strongest genotype-phenotype association described so far for MS [34]. Therefore, the differences in genetic load driven by the age of onset quantitative trait loci suggest that the two groups (high P-Hat and misclassified) are characterized by overlapping but distinct genetic profiles.

### Functional annotation enrichment

To gain insights into the biological significance of the 350 variants identified in our analysis and assess how these may relate to the etiology of MS, we interrogated the gene list for enrichment of known biological labels such as gene ontologies and protein pathways. DAVID [31] identified significant enrichment for ontological categories relating to cell adhesion, cell communication/signaling, and development (Table 6). Pathway Express identified significant enrichment of the KEGG (Kyoto Encyclopedia of Genes and Genomes) pathways for cell adhesion molecules, neuroactive ligand-receptor interactions, allograft rejection, and type I diabetes mellitus, including well-defined immunological genes coding for adhesive molecules (*CD58*, *CD226*, *SELPLG*, and *VCAM1*) and MHC class I and class II genes.

**Table 6:** Functional annotation of the 350 genes

Gene Ontology^a^	DAVID^b^
**Biological process**	
Cell adhesion (GO:0007155)	0.0000148
Cell communication	0.000632
G-protein signaling, coupled to cyclic nucleotide second messenger	0.001940^c^
System development (GO:0048731)	0.000000016
Central nervous system development	0.000293^c^
Organ development (GO:0048513)	0.000017
	
**Cellular compartment**	
Integral to membrane (GO:0016021)	0.0000018
Integral to plasma membrane (GO:0005887)	0.000000026
Dystrophin-associated glycoprotein complex	0.002081^c^
Sarcoglycan complex	0.004398^c^
	
**Molecular function**	
Signal transducer activity (GO:0004871)	0.0000025
Transmembrane receptor activity (GO:0004888)	0.0000274
Transmembrane receptor protein phosphatase activity	0.003811^c^
Amine receptor activity	0.004557^c^
Hematopoietin/interferon-class (D200-domain) cytokine receptor activity	0.001526^c^
Phosphoinositide binding	0.000737^c^
GPI anchor binding	0.003257^c^
Calcium-release channel activity	0.004102^c^
Delayed rectifier potassium channel activity	0.001212^c^
	
**Enriched KEGG pathways**	PE^b^
Cell adhesion molecules (CAMs)	0.00000036
Neuroactive ligand-receptor interaction	0.000542
Allograft rejection	0.001545
Type I diabetes mellitus	0.003487

^a^Only significant Gene Ontology levels 4 or higher are indicated for clarity. ^b^*P*-value correction: DAVID, Benjamini; Pathway Express (PE), FDR. ^c^Analysis results using GOTree Machine [32]. KEGG, Kyoto Encyclopedia of Genes and Genomes.

## Discussion

Partially powered GWAS and ensuing meta-analysis have identified a number of non-HLA candidate genes associated with MS susceptibility [11-14]. Each significant association has a very modest effect, representing a small share of the genetic variance affecting disease risk. In this follow-up study of the meta-analysis dataset, we applied logistic regression stepwise selection methods and identified 350 variants. We used these markers to build a genetic profile associated with the cumulative genetic risk measured by the probability of an individual being a MS case. In the validation dataset, we tested the model and found that the classification algorithm yielded 62.3% sensitivity and 75.9% specificity, with an AUC of 0.769. These numbers together indicate that the application of the genetic profile built using the meta-analysis discovery dataset does not provide a high discriminatory accuracy in the independent dataset despite a median cumulative genetic risk in the discovery dataset of 0.90 for the case group, and 0.01 for the control group. For the validation dataset, the values are 0.59 for the case group and 0.32 for the control group.

**Figure 3:**
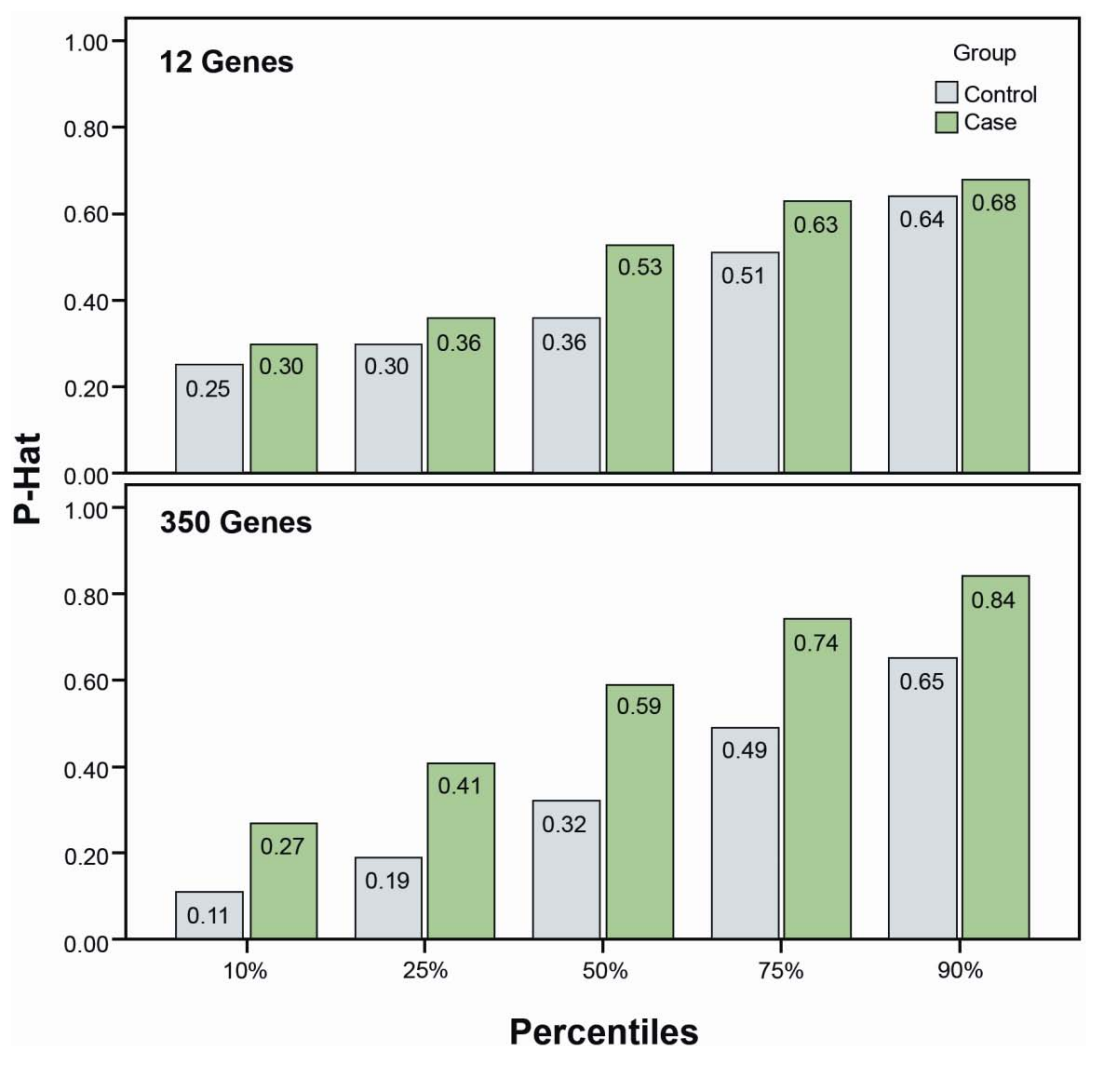
**Distribution of the estimated cumulative genetic risk (P-Hat) of case and control groups using the 12-gene set and 350-gene set in the validation dataset**. P-Hat is the estimated cumulative genetic risk (the probability of being a MS case). The median of the cumulative genetic risk (50% quantile) in the case group is 0.59, and in the control group 0.32. The genetic profile produced a significant difference of P-Hat between the case and control groups.

In order to better understand the magnitude of variance explained by different sets of genes in the logistic regression models, adjusted R^2 ^(Nagelkerke's R^2^) of different models using the discovery and validation datasets were compared (summarized in Table 7). This analysis assigns to the HLA-DRB1*15:01 allele approximately 7% of the total variance in the predictive model. The 11 validated genes explain about 3% of the remaining variance in the discovery dataset and 2% in the validation dataset. For the 350-gene set, the 349 genes in addition to *HLA-DRB1 *in the model explain 49% and 17% of the total variance in the discovery and validation datasets, respectively. The estimated cumulative genetic risk in the validation dataset using the 12 validated genes did not show significant differences between the case and control groups (Figure 3). On the other hand, the 350-gene set contributed to improved classification sensitivity, from 54.3% (12 genes) to 62.3% (350 genes) in the validation process (Table 4). Furthermore, when using only the 12 genes, all DRB1*15:01-negative individuals in the validation dataset were classified as controls, which explains the higher specificity observed in the 12-gene-set models and its lack of discriminatory power for DRB1*15:01-negative individuals. Finally, the 350-gene set includes 6 markers in the MHC region other than *DRB1*, and these are associated with the largest observed *P*-values. In order to assess if they play a surrogate role when calculating the cumulative genetic risk (P-Hat) in the genetic profile, we used logistic regression condition on DRB1*15:01 (+/-) to assess R^2 ^of the six MHC variants. The total variance accounted for these non-*DRB1 *MHC genes is 2.1% in the discovery dataset, and 2.6% in the replication dataset.

**Table 7:** The percentage of variance (R^2^) explained by predictors in the regression model

	Center	Gender	DRB1*15:01	12 genes^a^	350 genes^b^
The discovery dataset (n = 8,844)	15%	4%	7%	10%	57%
The validation dataset (n = 3,606)	NA	2%	9%	11% (AUC^c ^= 0.68)	27% (AUC^c ^= 0.769)

^a^The 12-gene set includes *HLA-DRB1 *and 11 additional validated genes. ^b^The 350-gene set includes *HLA-DRB1 *and 349 additional genes identified in the genetic profile. ^c^AUC, area under curve from ROC analysis results.

Several factors could have contributed to the relatively low sensitivity of the selected genes. First, the power of the discovery dataset is more likely inadequate to detect all susceptibility genes. Even though we have used the largest MS genetic dataset available to date, it has been suggested that a dataset with 10,000 cases and 10,000 controls might be able to reach a desirable level of power for GWAS analysis in order to effectively control both type I and type II errors. This is especially valid for less frequent alleles (minor allele frequency ≤10%) and effect size (odds ratio) in the range 1.1 to 1.3 [35,36]. Second, relevant MS variants may have gone undetected because of the partial genome coverage in the currently available SNP arrays. Third, there are unknown interactions between genes involved in the biochemical pathways that contribute to MS susceptibility. Fourth, the total adjusted R^2 ^of the logistic regression model is 0.75 and the r-square attributable to genetic factors in this model accounted for only 56.5%, suggesting that without fitting environmental triggers into the model, predictive accuracy will remain limited. A large number of environmental exposures have been investigated in MS, but recent epidemiologic and laboratory studies have provided support primarily for vitamin D and Epstein-Barr virus exposure [37,38]. A recent study suggests that adding environmental risk factors into a predictive algorithm based on genetic variants enhances the case-control status classification [39]. Fifth, due to the suboptimal power in the discovery dataset, it is likely that the selected 350 variants include both true and false signals. The inclusion of false positives in the estimators that fit the discovery dataset does not contribute to the prediction in the validating process, also causing a tractable drop in classification accuracy. Thus, the results shown in Table 4 may contain a portion of overestimation of model fit in the discovery dataset analysis results, indicating that bias could be embedded in predictive modeling when using the association tests approach in marker selection.

All these confounders are reflected in the fact that some individuals in the control group carry a high cumulative genetic risk (P-Hat >0.8). Thus, in this experiment utilizing the most updated MS genetic dataset, a high cumulative genetic risk is not sufficient to predict with high confidence affectation status even in the discovery dataset (Table 4). Additional layers of complexity are represented by the likelihood of unaccountable epistatic interactions, etiological heterogeneity, and epigenetic and random events. These limitations notwithstanding, the genetic risk as assessed here still captures a significant portion of the full cumulative genetic risk (the probability of being a MS case) in the validation dataset between the case (median = 0.59, 75% quartile = 0.74) and control group (median = 0.32, 75% quartile = 0.49). The model with the 350-gene set produced a larger difference of the estimated cumulative genetic risk between case and control groups compared with that produced by the 12-gene set in the models (Figure 3). Thus, the cumulative genetic risk (P-Hat) generated using the 350-gene set can still provide a useful index of the genetic load associated with MS, and provides important mechanistic insights.

Most validated MS susceptibility loci have well-defined roles in immunologic functions, consistent with the hypothesis that MS etiology has its primary roots in early immune system dysregulation, precipitating secondary neuronal degeneration. On the other hand, a network-based pathway analysis of two GWAS in MS, where evidence for genetic association was combined with evidence for protein-protein interaction, demonstrated the role of neural pathway genes (axon guidance and long-term potentiation) in conferring susceptibility [26]. The genetic profile identified in this analysis confirms the significant enrichment of genes involved not only in the immune response but also in nervous system development and neuronal signaling (Table 6). These included genes encoding cell-cell adhesion molecules (*CDH2*, *CADM1*, *CNTN1*, *NCAM2*, *NRXN1*, and *NRXN3*) and several neuronal receptors, such as the G-protein coupled receptors (*ADRA1A*, *ADARA2A*, *GABRB3*, *TACR1*, *CHR3*, *HTR1B*, *HTR1E*, and *HTR2A*), as well as the metabotropic glutamate receptor (*GRM8*) and ionotropic glutamate receptors (*GRIK4 *and *GRIN2B*). Interestingly, members of the glutamate receptor pathway have been previously identified by our group in both the network-based study of one of the GWAS datasets included in the meta-analysis utilized here (*GRIN2A*, *GRIK1*, *GRIK2*, *GRIK4*, *GRID2*, *GRIA1*, *GRIK4*) [26] and an independent pharmacogenomic study of type I interferon response (*GRIA1*, *GRID2*, *SLC1A2*) [40]. A more recent pharmacogenomic study also identified the ionotropic glutamate receptor (*GRIA3*) associated with interferon response in MS [41]. These observations further support the proposed mechanism of glutamate excitotoxicity as a precipitating agent of the glial and axonal injury observed in MS [42,43]. The ramifications of these SNPs on expression or function are unknown; however, their recent and continued identification may help evolve a model of MS pathogenesis with increasing contributions from neuronal genes.

In summary, the cumulative genetic risk estimation using a genetic profile composed of 350 genes provides a useful index of the genetic risk leading to MS. The incomplete classification accuracy reflects most likely the limited power of available genetic datasets and the difficulties in incorporating gene-gene interactions and gene-environment interactions. The imminent publication of larger high-resolution GWAS and transcriptomic studies together with recent progress in identifying true environmental variables will refine this and other modeling approaches for a greater understanding of MS genetics and assessment of translational applications.

## Abbreviations

ANCOVA: analysis of covariance; AUC: area under curve; FDR: false discovery rate; GWAS: genome-wide association study; HLA: human leukocyte antigen; LD: linkage disequilibrium; MS: multiple sclerosis; MSSS: Multiple Sclerosis Severity Score; ROC: receiver operating characteristic; SNP: single-nucleotide polymorphism.

## Competing interests

The authors declare that they have no competing interests.

## Authors' contributions

JW and JRO conceived and designed the experiments. JW, PIWdB and PLdJ performed the experiments. JW completed the statistical analysis. SEB, DP, DP, LBC, PIWdB, LK, CHP, DAH and PLdJ contributed reagents/materials/analysis tools. JW, JRO, DP and PMM wrote the paper.

## Supplementary Material

Additional file 1 **Table S1**. Marker information of the 12 validated genes. Click here for file

Additional file 2 **Table S2**. Flow chart of analysis procedures to identify independent MS susceptibility markers. Click here for file

Additional file 3 **Table S3**. Independent markers significant at FDR *P *≤ 0.05 in the discovery dataset (N = 8,844). Click here for file

Additional file 4 **Table S4**. Genetic profile used for assessing the cumulative genetic risk (350 genes). Click here for file
