# Supplementary material for: Modeling the cumulative genetic risk for multiple sclerosis from genome-wide association data
Source: Genome Med. 2011 Jan 18;3(1):3. doi: 10.1186/gm217 (PMC3092088; doi:10.1186/gm217)
Supplement: Additional file 1 — Table S1. Marker information of the 12 validated genes. [file gm217-S1.DOC]

Table S1.

Marker information of the 12 validated genes.

| **RSID** | **GeneName** | **Chrom** | **Allele** | **RiskAllele** | **MAF** | **OR-Risk Allele** |
| --- | --- | --- | --- | --- | --- | --- |
| rs12708716 | CLEC16A | 16 | G,A | A | 0.34 | 1.16 |
| rs12720307 | TYK2 | 19 | T,C | T | 0.14 | 1.16 |
| rs17445836 | IRF8 | 16 | A,G | G | 0.20 | 1.24 |
| rs17824933 | CD6 | 11 | G,C | G | 0.24 | 1.16 |
| rs1800693 | TNFRSF1A | 12 | C,T | C | 0.43 | 1.22 |
| rs2104286 | IL2RA | 10 | C,T | T | 0.24 | 1.16 |
| rs2300747 | CD58 | 1 | G,A | A | 0.12 | 1.29 |
| rs3135388 | HLA-DRB1 | 6 | A,G | A | 0.21 | 2.77 |
| rs6897932 | IL7R | 5 | T,C | C | 0.26 | 1.12 |
| rs7237611 | CD226 | 18 | G,A | G | 0.30 | 1.13 |
| rs727986 | GPC5 | 13 | G,A | A | 0.26 | 1.10 |
| rs11164814 | EVI5 | 1 | A,G | A | 0.27 | 1.20 |
