# Supplementary material for: Modeling the cumulative genetic risk for multiple sclerosis from genome-wide association data
Source: Genome Med. 2011 Jan 18;3(1):3. doi: 10.1186/gm217 (PMC3092088; doi:10.1186/gm217)
Supplement: Additional file 2 — Table S2. Flow chart of analysis procedures to identify independent MS susceptibility markers. [file gm217-S2.DOC]

Table S2.

Flow chart of analysis procedures to identify independent MS susceptibility markers.

1. Logistic regression conditioning on *DRB1*1501* status (+/-), with covariates center and gender.

--selected the top significant markers aat at p < 10^-5^

2a. Logistic regression type I test on the selected set of markers to examine independence

2b. Logistic regression type III test on the selected set of markers to examine independence

--top significant independent markers are identified (p<0.01 from both tests)

--select the second set of significant markers at p<0.001

4a. Logistic regression type I test on the selected set of markers to examine independence

4b. Logistic regression type III test on the selected set of markers to examine independence

--top significant independent markers are identified (p<0.01 from both tests)

--select the third set of significant markers at p<0.001

Procedure continues until all markers with an independent effect at p<0.001 are identified.

3. Place this set of markers into a logistic regression model (type III test) to search for markers with remaining effect at p<0.001.

5. Place the first and second sets of markers into a logistic regression model (type III test) to search for markers with remaining effect at p<0.001.
