# Supplementary material for: Modeling the cumulative genetic risk for multiple sclerosis from genome-wide association data
Source: Genome Med. 2011 Jan 18;3(1):3. doi: 10.1186/gm217 (PMC3092088; doi:10.1186/gm217)
Supplement: Additional file 3 — Table S3. Independent markers significant at FDR P ≤ 0.05 in the discovery dataset (N = 8,844). [file gm217-S3.DOC]

Table S3.

Independent markers significant at FDR p≤0.05 in the discovery dataset (N=8844)

| **RSID** | **Position** | **Chrom** | **Gene Name** | **A1** | **A2** | **-Log10 p** | **OR** | **LowerCL** | **UpperCL** |
| --- | --- | --- | --- | --- | --- | --- | --- | --- | --- |
| rs9268148 | 32367505 | 6 | C6orf10 | A | G | 13.13 | 0.58 | 0.50 | 0.67 |
| rs1611715 | 29937461 | 6 | HLA-G | C | A | 11.49 | 0.74 | 0.68 | 0.81 |
| rs7772297 | 31436805 | 6 | HLA-B | C | G | 9.14 | 1.40 | 1.26 | 1.56 |
| rs4939490 | 60550227 | 11 | CD6 | G | C | 9.00 | 1.30 | 1.19 | 1.42 |
| rs9275596 | 32789609 | 6 | HLA-DQA2 | T | C | 7.85 | 0.76 | 0.69 | 0.84 |
| rs10244467 | 22584456 | 7 | IL6 | T | C | 7.23 | 0.57 | 0.47 | 0.70 |
| rs9596270 | 49740441 | 13 | DLEU1 | T | C | 7.08 | 1.56 | 1.31 | 1.85 |
| rs12025416 | 116750329 | 1 | CD58 | C | T | 6.83 | 0.69 | 0.59 | 0.80 |
| rs6836440 | 100405684 | 4 | ADH4 | A | G | 6.74 | 0.68 | 0.58 | 0.79 |
| rs7137953 | 119357405 | 12 | GATC | C | T | 6.47 | 0.77 | 0.70 | 0.85 |
| rs10846336 | 16413619 | 12 | MGST1 | T | C | 6.43 | 0.42 | 0.30 | 0.59 |
| rs931555 | 35839334 | 5 | IL7R | C | T | 6.41 | 1.25 | 1.15 | 1.36 |
| rs10203141 | 179015804 | 2 | OSBPL6 | C | G | 6.40 | 0.81 | 0.75 | 0.88 |
| rs2328523 | 20575342 | 6 | E2F3 | G | A | 6.28 | 0.79 | 0.72 | 0.87 |
| rs4368946 | 98497864 | 8 | TSPYL5 | T | C | 6.25 | 0.70 | 0.61 | 0.80 |
| rs3934035 | 281714 | 3 | CHL1 | C | T | 6.23 | 0.46 | 0.34 | 0.62 |
| rs17062281 | 73654880 | 13 | KLF12 | C | G | 6.13 | 0.44 | 0.31 | 0.61 |
| rs1356122 | 155666264 | 3 | GPR149 | G | C | 6.13 | 1.26 | 1.14 | 1.40 |
| rs4447 | 31599694 | 22 | SYN3 | T | C | 6.10 | 0.74 | 0.66 | 0.83 |
| rs655763 | 108682027 | 11 | C11orf87 | C | T | 6.03 | 1.59 | 1.32 | 1.92 |
| rs12419184 | 125561518 | 11 | RPUSD4 | C | T | 6.03 | 0.72 | 0.63 | 0.82 |
| rs426921 | 63402133 | 18 | DSEL | C | T | 5.89 | 1.28 | 1.14 | 1.43 |
| rs2104286 | 6139051 | 10 | IL2RA | T | C | 5.88 | 1.24 | 1.13 | 1.36 |
| rs1000361 | 27785323 | 3 | EOMES | A | G | 5.84 | 0.83 | 0.76 | 0.90 |
| rs7349811 | 96513963 | 5 | LIX1 | T | C | 5.80 | 1.20 | 1.11 | 1.30 |
| rs1182580 | 104158803 | 1 | AMY1C | C | T | 5.68 | 1.21 | 1.12 | 1.31 |
| rs395561 | 106889874 | 5 | EFNA5 | A | C | 5.65 | 0.83 | 0.77 | 0.90 |
| rs7299943 | 122118365 | 12 | PITPNM2 | T | A | 5.64 | 0.80 | 0.73 | 0.88 |
| rs6816551 | 42934347 | 4 | GRXCR1 | G | A | 5.64 | 0.80 | 0.73 | 0.88 |
| rs724326 | 136319703 | 2 | R3HDM1 | T | C | 5.62 | 0.79 | 0.72 | 0.87 |
| rs7247028 | 16716990 | 19 | NWD1 | A | G | 5.61 | 0.71 | 0.62 | 0.82 |
| rs10146906 | 23603379 | 14 | CPNE6 | C | A | 5.60 | 1.30 | 1.16 | 1.45 |
| rs1800454 | 32908390 | 6 | TAP2 | C | T | 5.59 | 1.37 | 1.20 | 1.56 |
| rs2045768 | 38544301 | 4 | KLF3 | G | C | 5.59 | 1.22 | 1.12 | 1.33 |
| rs9361086 | 77354622 | 6 | IMPG1 | C | G | 5.59 | 1.46 | 1.25 | 1.71 |
| rs7634068 | 30221941 | 3 | RBMS3 | T | G | 5.58 | 0.68 | 0.57 | 0.80 |
| rs2900741 | 32400723 | 11 | WT1 | G | A | 5.58 | 0.81 | 0.75 | 0.89 |
| rs528438 | 23927873 | 18 | CDH2 | T | C | 5.56 | 0.78 | 0.70 | 0.86 |
| rs1436665 | 57633331 | 12 | LRIG3 | G | A | 5.54 | 0.77 | 0.69 | 0.86 |
| rs12954376 | 57035772 | 18 | CDH20 | T | A | 5.54 | 0.82 | 0.75 | 0.89 |
| rs9931083 | 77856003 | 16 | WWOX | C | T | 5.54 | 0.54 | 0.42 | 0.70 |
| rs434496 | 86685205 | 8 | REXO1L1 | T | C | 5.51 | 0.79 | 0.71 | 0.87 |
| rs4737206 | 66292544 | 8 | ARMC1 | C | T | 5.50 | 1.38 | 1.20 | 1.57 |
| rs323242 | 77759252 | 6 | HTR1B | G | A | 5.46 | 0.82 | 0.75 | 0.89 |
| rs9497275 | 145728075 | 6 | UTRN | T | C | 5.45 | 0.46 | 0.33 | 0.64 |
| rs9586741 | 104537871 | 13 | G30 | A | C | 5.44 | 1.23 | 1.13 | 1.34 |
| rs7296395 | 79087750 | 12 | C12orf64 | C | T | 5.43 | 0.60 | 0.48 | 0.76 |
| rs9469446 | 33592997 | 6 | BAK1 | C | G | 5.42 | 0.37 | 0.24 | 0.56 |
| rs10518326 | 120036902 | 4 | SEC24D | G | A | 5.41 | 0.69 | 0.59 | 0.81 |
| rs11065987 | 110535144 | 12 | BRAP | A | G | 5.40 | 0.83 | 0.76 | 0.90 |
| rs4863704 | 141187379 | 4 | MAML3 | T | C | 5.39 | 0.59 | 0.48 | 0.74 |
| rs3806010 | 70847640 | 6 | COL19A1 | C | T | 5.37 | 0.72 | 0.63 | 0.83 |
| rs11755663 | 13260894 | 6 | PHACTR1 | T | C | 5.35 | 0.80 | 0.72 | 0.88 |
| rs17765606 | 64540948 | 16 | CDH5 | A | G | 5.34 | 0.60 | 0.48 | 0.74 |
| rs7789322 | 127247763 | 7 | SND1 | C | T | 5.33 | 1.21 | 1.12 | 1.32 |
| rs2726485 | 106621054 | 4 | PPA2 | T | G | 5.31 | 0.84 | 0.78 | 0.91 |
| rs6896969 | 40460183 | 5 | PTGER4 | C | A | 5.28 | 1.20 | 1.11 | 1.30 |
| rs4674841 | 224704922 | 2 | SERPINE2 | G | T | 5.28 | 0.39 | 0.26 | 0.59 |
| rs10815231 | 547015 | 9 | KANK1 | T | G | 5.28 | 0.81 | 0.73 | 0.89 |
| rs8187919 | 72774351 | 9 | ALDH1A1 | A | G | 5.27 | 0.58 | 0.46 | 0.73 |
| rs10885868 | 117912102 | 10 | GFRA1 | T | C | 5.26 | 1.20 | 1.11 | 1.30 |
| rs1144575 | 176579059 | 1 | TOR1AIP1 | G | T | 5.25 | 0.81 | 0.73 | 0.89 |
| rs10278177 | 152091151 | 7 | ACTR3B | G | A | 5.21 | 1.34 | 1.18 | 1.53 |
| rs10762363 | 71734104 | 10 | LRRC20 | A | C | 5.19 | 0.83 | 0.76 | 0.90 |
| rs13031248 | 236499409 | 2 | CENTG2 | G | A | 5.17 | 0.35 | 0.22 | 0.55 |
| rs13395283 | 23185280 | 2 | KLHL29 | T | G | 5.15 | 0.63 | 0.51 | 0.77 |
| rs595985 | 137968020 | 6 | TNFAIP3 | T | C | 5.12 | 1.24 | 1.13 | 1.37 |
| rs17803373 | 85849951 | 6 | RPL31P32 | T | G | 5.12 | 1.44 | 1.23 | 1.68 |
| rs7604003 | 15897703 | 2 | DDX1 | G | A | 5.11 | 0.83 | 0.76 | 0.90 |
| rs4447946 | 50469540 | 5 | ISL1 | T | A | 5.09 | 0.69 | 0.58 | 0.81 |
| rs927544 | 46354052 | 13 | HTR2A | A | G | 5.06 | 1.22 | 1.12 | 1.33 |
| rs1432205 | 137729996 | 2 | THSD7B | A | C | 5.06 | 0.82 | 0.76 | 0.90 |
| rs4740392 | 130894500 | 9 | LAMC3 | G | A | 5.05 | 1.32 | 1.16 | 1.52 |
| rs12960174 | 75693795 | 18 | KCNG2 | T | C | 5.05 | 0.81 | 0.74 | 0.89 |
| rs688585 | 206889899 | 1 | HHAT | A | T | 5.03 | 1.20 | 1.11 | 1.30 |
| rs17086658 | 57010834 | 4 | KIAA1211 | C | T | 4.99 | 1.59 | 1.28 | 1.96 |
| rs11870121 | 71370533 | 17 | WBP2 | A | G | 4.99 | 0.80 | 0.73 | 0.89 |
| rs9901869 | 42930205 | 17 | NPEPPS | A | G | 4.94 | 0.84 | 0.78 | 0.91 |
| rs6691787 | 68118647 | 1 | GNG12 | C | T | 4.93 | 0.82 | 0.74 | 0.89 |
| rs2492853 | 88061478 | 9 | ISCA1 | T | C | 4.93 | 0.84 | 0.76 | 0.92 |
| rs13029809 | 32859845 | 2 | TTC27 | A | G | 4.91 | 0.72 | 0.62 | 0.83 |
| rs2515585 | 6316818 | 8 | MCPH1 | C | A | 4.90 | 0.79 | 0.71 | 0.88 |
| rs3098171 | 48558803 | 15 | USP8 | G | C | 4.89 | 1.19 | 1.10 | 1.29 |
| rs7710308 | 85465217 | 5 | COX7C | G | A | 4.88 | 1.19 | 1.10 | 1.29 |
| rs9924445 | 85304319 | 16 | FOXL1 | G | A | 4.87 | 0.38 | 0.25 | 0.59 |
| rs10150582 | 87782541 | 14 | KCNK10 | A | G | 4.87 | 0.54 | 0.41 | 0.71 |
| rs750780 | 33874170 | 11 | LMO2 | G | A | 4.86 | 0.78 | 0.70 | 0.87 |
| rs4932507 | 89976286 | 15 | TRNAY16P | G | A | 4.86 | 0.83 | 0.76 | 0.90 |
| rs3845915 | 124822524 | 3 | MYLK | A | G | 4.86 | 0.78 | 0.70 | 0.87 |
| rs1050779 | 56637716 | 16 | MMP15 | C | G | 4.86 | 0.80 | 0.72 | 0.88 |
| rs2233263 | 46880261 | 3 | MYL3 | C | T | 4.85 | 0.57 | 0.44 | 0.73 |
| rs10051942 | 174043508 | 5 | MSX2 | G | C | 4.85 | 1.24 | 1.13 | 1.37 |
| rs4708970 | 162996789 | 6 | PARK2 | T | C | 4.85 | 0.79 | 0.71 | 0.88 |
| rs4782888 | 82651198 | 16 | MBTPS1 | A | C | 4.85 | 0.64 | 0.52 | 0.78 |
| rs2841309 | 100858611 | 6 | SIM1 | C | T | 4.85 | 0.84 | 0.78 | 0.91 |
| rs12711507 | 208199962 | 1 | NEK2 | A | G | 4.84 | 0.76 | 0.67 | 0.86 |
| rs11196056 | 114435698 | 10 | VTI1A | A | G | 4.84 | 0.76 | 0.68 | 0.86 |
| rs6837662 | 79856615 | 4 | ANXA3 | A | G | 4.81 | 3.43 | 1.96 | 6.00 |
| rs2713768 | 102225451 | 3 | ABI3BP | A | T | 4.80 | 0.79 | 0.71 | 0.88 |
| rs238342 | 41716466 | 13 | DGKH | C | T | 4.79 | 0.84 | 0.78 | 0.91 |
| rs6463156 | 4703519 | 7 | MMD2 | A | G | 4.79 | 1.22 | 1.12 | 1.34 |
| rs11769998 | 25244176 | 7 | NPVF | T | C | 4.79 | 0.70 | 0.59 | 0.82 |
| rs17103790 | 19743825 | 14 | OR11H5P | T | G | 4.78 | 0.79 | 0.71 | 0.88 |
| rs8078184 | 46020143 | 17 | CACNA1G | A | G | 4.78 | 1.20 | 1.11 | 1.31 |
| rs11157866 | 51441063 | 14 | GNG2 | C | T | 4.78 | 0.78 | 0.69 | 0.88 |
| rs9535449 | 49962302 | 13 | DLEU7 | T | C | 4.76 | 0.74 | 0.65 | 0.85 |
| rs6576873 | 87383694 | 1 | HS2ST1 | T | C | 4.71 | 2.74 | 1.72 | 4.36 |
| rs698853 | 53965523 | 2 | ASB3 | A | G | 4.71 | 1.21 | 1.11 | 1.32 |
| rs17445836 | 84575164 | 16 | IRF8 | A | G | 4.71 | 0.80 | 0.73 | 0.89 |
| rs7187161 | 12602404 | 16 | SNX29 | C | T | 4.70 | 0.85 | 0.78 | 0.91 |
| rs11618516 | 39095832 | 13 | LHFP | A | G | 4.69 | 0.80 | 0.72 | 0.88 |
| rs10271373 | 138187050 | 7 | ZC3HAV1 | C | A | 4.69 | 0.87 | 0.80 | 0.94 |
| rs11264082 | 37791105 | 1 | RSPO1 | G | A | 4.68 | 1.35 | 1.17 | 1.54 |
| rs3794716 | 78152456 | 17 | FOXK2 | G | A | 4.68 | 0.69 | 0.59 | 0.82 |
| rs7814314 | 136905069 | 8 | KHDRBS3 | G | A | 4.67 | 0.81 | 0.74 | 0.89 |
| rs10999626 | 72418416 | 10 | PCBD1 | A | G | 4.64 | 0.81 | 0.74 | 0.89 |
| rs2758457 | 114311501 | 6 | HDAC2 | C | T | 4.64 | 0.50 | 0.36 | 0.71 |
| rs6702388 | 161702018 | 1 | PBX1 | T | C | 4.64 | 1.45 | 1.22 | 1.72 |
| rs7314705 | 30090640 | 12 | TMTC1 | T | C | 4.63 | 1.53 | 1.26 | 1.86 |
| rs7289336 | 25967472 | 22 | MIAT | A | G | 4.63 | 1.59 | 1.28 | 1.97 |
| rs199138 | 43174842 | 15 | DUOX2 | G | A | 4.63 | 1.83 | 1.38 | 2.41 |
