# Supplementary material for: Modeling the cumulative genetic risk for multiple sclerosis from genome-wide association data
Source: Genome Med. 2011 Jan 18;3(1):3. doi: 10.1186/gm217 (PMC3092088; doi:10.1186/gm217)
Supplement: Additional file 4 — Table S4. Genetic profile used for assessing the cumulative genetic risk (350 genes). [file gm217-S4.DOC]

Table S4.

Genetic profile used for assessing the cumulative genetic risk (350 genes)

| RSID | Position | Chrom | Gene Name | A1 | A2 | HighLP | OR | LowerCL | UpperCL |
| --- | --- | --- | --- | --- | --- | --- | --- | --- | --- |
| rs3748816 | 2558908 | 1 | MMEL1 | G | A | 3.28 | 0.86 | 0.79 | 0.94 |
| rs1203628 | 13816132 | 1 | PRDM2 | T | A | 3.80 | 1.48 | 1.21 | 1.82 |
| rs1193345 | 17659020 | 1 | ARHGEF10L | A | G | 4.28 | 1.70 | 1.31 | 2.20 |
| rs10916665 | 19960300 | 1 | OTUD3 | T | A | 4.25 | 0.85 | 0.79 | 0.92 |
| rs11811702 | 33019276 | 1 | HPCA | A | G | 4.31 | 1.20 | 1.09 | 1.31 |
| rs6661842 | 33940961 | 1 | CSMD2 | C | T | 4.13 | 0.85 | 0.78 | 0.92 |
| rs12145367 | 39295431 | 1 | MACF1 | G | C | 3.01 | 1.16 | 1.06 | 1.27 |
| rs1002357 | 53465917 | 1 | LRP8 | G | C | 3.16 | 1.30 | 1.10 | 1.53 |
| rs11580092 | 58546047 | 1 | DAB1 | A | G | 3.22 | 1.13 | 1.05 | 1.23 |
| rs17118258 | 59013168 | 1 | JUN | G | A | 4.07 | 0.46 | 0.31 | 0.68 |
| rs17379082 | 77524824 | 1 | AK5 | A | G | 4.35 | 0.81 | 0.74 | 0.90 |
| rs6576873 | 87383694 | 1 | HS2ST1 | T | C | 4.71 | 2.74 | 1.72 | 4.36 |
| rs11165441 | 91936368 | 1 | TGFBR3 | G | A | 4.18 | 1.25 | 1.12 | 1.39 |
| rs11164814 | 92973394 | 1 | EVI5 | A | G | 4.25 | 0.84 | 0.76 | 0.91 |
| rs17124182 | 101487272 | 1 | S1PR1 | G | C | 3.12 | 0.67 | 0.53 | 0.85 |
| rs4839309 | 113583045 | 1 | MAGI3 | G | C | 4.51 | 0.72 | 0.62 | 0.84 |
| rs12025416 | 116750329 | 1 | CD58 | C | T | 6.83 | 0.69 | 0.59 | 0.80 |
| rs17659359 | 149351431 | 1 | LCE3E | C | G | 3.70 | 1.20 | 1.09 | 1.31 |
| rs1144575 | 176579059 | 1 | TOR1AIP1 | G | T | 5.25 | 0.81 | 0.73 | 0.89 |
| rs2760524 | 189262205 | 1 | RGS1 | G | A | 3.98 | 1.23 | 1.10 | 1.36 |
| rs7522462 | 197613252 | 1 | KIF21B | G | A | 4.49 | 1.20 | 1.10 | 1.31 |
| rs688585 | 206889899 | 1 | HHAT | A | T | 5.03 | 1.20 | 1.11 | 1.30 |
| rs12711507 | 208199962 | 1 | NEK2 | A | G | 4.84 | 0.76 | 0.67 | 0.86 |
| rs906363 | 209247192 | 1 | BATF3 | T | C | 3.18 | 1.19 | 1.07 | 1.33 |
| rs2841346 | 235272587 | 1 | CHRM3 | T | C | 4.03 | 1.17 | 1.08 | 1.27 |
| rs12035032 | 238989648 | 1 | PLD5 | C | T | 3.06 | 1.15 | 1.06 | 1.25 |
| rs1720971 | 7772816 | 2 | RNF144 | T | C | 4.15 | 1.21 | 1.10 | 1.33 |
| rs13406824 | 9839643 | 2 | YWHAQ | C | G | 3.61 | 0.85 | 0.78 | 0.93 |
| rs1016567 | 15263939 | 2 | NAG | T | C | 3.64 | 1.18 | 1.08 | 1.28 |
| rs7604003 | 15897703 | 2 | DDX1 | G | A | 5.11 | 0.83 | 0.76 | 0.90 |
| rs11691181 | 18039500 | 2 | KCNS3 | G | T | 3.47 | 0.73 | 0.60 | 0.88 |
| rs1437861 | 19931571 | 2 | FLJ12334 | G | A | 4.25 | 0.85 | 0.79 | 0.92 |
| rs13398690 | 29781044 | 2 | ALK | A | C | 3.05 | 0.72 | 0.59 | 0.88 |
| rs6718520 | 43237221 | 2 | HAAO | G | A | 3.01 | 0.89 | 0.82 | 0.96 |
| rs11893174 | 49893639 | 2 | FSHR | C | G | 4.27 | 0.71 | 0.60 | 0.85 |
| rs6545242 | 52055919 | 2 | NRXN1 | G | A | 3.97 | 0.85 | 0.78 | 0.92 |
| rs698853 | 53965523 | 2 | ASB3 | A | G | 4.71 | 1.21 | 1.11 | 1.32 |
| rs4672121 | 56641018 | 2 | CCDC85A | G | A | 3.78 | 0.86 | 0.80 | 0.93 |
| rs2160654 | 75322712 | 2 | TACR1 | T | C | 4.28 | 0.83 | 0.76 | 0.91 |
| rs4583488 | 104478109 | 2 | POU3F3 | G | A | 3.22 | 2.22 | 1.41 | 3.51 |
| rs11686294 | 112186938 | 2 | ANAPC1 | A | G | 3.36 | 1.15 | 1.06 | 1.24 |
| rs1866657 | 127157388 | 2 | GYPC | A | G | 4.20 | 1.20 | 1.10 | 1.31 |
| rs724326 | 136319703 | 2 | R3HDM1 | T | C | 5.62 | 0.79 | 0.72 | 0.87 |
| rs13021210 | 140144905 | 2 | MRPS18BP2 | G | A | 3.09 | 0.87 | 0.79 | 0.94 |
| rs7560006 | 158926840 | 2 | CCDC148 | T | C | 3.82 | 0.54 | 0.39 | 0.74 |
| rs10191601 | 204280876 | 2 | RAPH1 | C | T | 4.26 | 1.19 | 1.09 | 1.30 |
| rs3770205 | 222121265 | 2 | EPHA4 | C | A | 3.07 | 0.86 | 0.78 | 0.94 |
| rs4674841 | 224704922 | 2 | SERPINE2 | G | T | 5.28 | 0.39 | 0.26 | 0.59 |
| rs6710297 | 230983017 | 2 | SP140 | A | G | 3.12 | 0.86 | 0.78 | 0.94 |
| rs6738310 | 240874475 | 2 | MYEOV2 | G | A | 3.23 | 1.27 | 1.11 | 1.45 |
| rs1153465 | 3130665 | 3 | IL5RA | C | T | 4.17 | 1.23 | 1.11 | 1.36 |
| rs6793708 | 5255161 | 3 | EDEM1 | C | T | 3.26 | 0.82 | 0.74 | 0.92 |
| rs11710881 | 16474007 | 3 | RFTN1 | C | T | 3.27 | 0.82 | 0.73 | 0.93 |
| rs2117876 | 24545583 | 3 | THRB | C | T | 3.33 | 1.33 | 1.13 | 1.55 |
| rs934328 | 30682748 | 3 | TGFBR2 | A | G | 3.50 | 1.17 | 1.08 | 1.28 |
| rs2233263 | 46880261 | 3 | MYL3 | C | T | 4.85 | 0.57 | 0.44 | 0.73 |
| rs323868 | 51953367 | 3 | PARP3 | A | G | 4.28 | 0.35 | 0.21 | 0.58 |
| rs7614366 | 100979041 | 3 | COL8A1 | G | T | 3.88 | 1.23 | 1.11 | 1.37 |
| rs2713768 | 102225451 | 3 | ABI3BP | A | T | 4.80 | 0.79 | 0.71 | 0.88 |
| rs4234492 | 102378713 | 3 | IMPG2 | T | G | 3.40 | 0.86 | 0.79 | 0.94 |
| rs16853570 | 144619900 | 3 | SLC9A9 | T | C | 3.03 | 0.73 | 0.61 | 0.88 |
| rs7634891 | 151695396 | 3 | TSC22D2 | T | C | 3.66 | 0.86 | 0.79 | 0.93 |
| rs1356122 | 155666264 | 3 | GPR149 | G | C | 6.13 | 1.26 | 1.14 | 1.40 |
| rs13072919 | 164841200 | 3 | IL12A | C | T | 3.66 | 1.40 | 1.17 | 1.67 |
| rs1420472 | 170259028 | 3 | GOLPH4 | T | G | 4.25 | 1.18 | 1.09 | 1.27 |
| rs451194 | 172771949 | 3 | PLD1 | C | T | 3.36 | 0.86 | 0.79 | 0.94 |
| rs711986 | 195737350 | 3 | TMEM44 | G | C | 3.59 | 0.85 | 0.77 | 0.93 |
| rs16835950 | 4732936 | 4 | STX18 | G | A | 3.91 | 1.73 | 1.31 | 2.30 |
| rs9990875 | 28374803 | 4 | STIM2 | C | G | 3.73 | 1.57 | 1.24 | 1.99 |
| rs6816551 | 42934347 | 4 | GRXCR1 | G | A | 5.64 | 0.80 | 0.73 | 0.88 |
| rs11930830 | 52816017 | 4 | SPATA18 | T | A | 3.08 | 0.80 | 0.71 | 0.92 |
| rs17086658 | 57010834 | 4 | KIAA1211 | C | T | 4.99 | 1.59 | 1.28 | 1.96 |
| rs10517506 | 61232206 | 4 | LPHN3 | A | C | 3.87 | 0.75 | 0.64 | 0.87 |
| rs6837662 | 79856615 | 4 | ANXA3 | A | G | 4.81 | 3.43 | 1.96 | 6.00 |
| rs9985612 | 90949067 | 4 | SNCA | C | T | 4.12 | 1.24 | 1.11 | 1.38 |
| rs17023134 | 96461849 | 4 | UNC5C | C | G | 3.05 | 1.34 | 1.13 | 1.59 |
| rs6836440 | 100405684 | 4 | ADH4 | A | G | 6.74 | 0.68 | 0.58 | 0.79 |
| rs2726485 | 106621054 | 4 | PPA2 | T | G | 5.31 | 0.84 | 0.78 | 0.91 |
| rs9998653 | 110400032 | 4 | COL25A1 | C | T | 3.68 | 1.21 | 1.09 | 1.33 |
| rs12498985 | 126283038 | 4 | ANKRD50 | C | T | 4.17 | 0.31 | 0.18 | 0.55 |
| rs736733 | 138544957 | 4 | PCDH18 | G | T | 3.09 | 1.18 | 1.07 | 1.30 |
| rs13148425 | 156998900 | 4 | GUCY1A3 | C | T | 3.68 | 0.71 | 0.59 | 0.85 |
| rs2597122 | 183499882 | 4 | ODZ3 | C | G | 3.45 | 1.48 | 1.15 | 1.92 |
| rs13139939 | 189179777 | 4 | ZFP42 | T | C | 3.93 | 0.72 | 0.61 | 0.85 |
| rs11749678 | 214292 | 5 | PLEKHG4B | C | T | 3.37 | 1.30 | 1.11 | 1.52 |
| rs13354562 | 2164936 | 5 | IRX4 | C | T | 3.96 | 1.37 | 1.16 | 1.62 |
| rs467326 | 3193979 | 5 | CEI | T | C | 3.75 | 0.82 | 0.74 | 0.91 |
| rs10512874 | 6736000 | 5 | SRD5A1 | G | A | 3.49 | 1.15 | 1.07 | 1.25 |
| rs6554856 | 14707464 | 5 | EEF1AL11 | G | A | 3.13 | 0.77 | 0.66 | 0.90 |
| rs10065696 | 20434123 | 5 | NUP50P3 | G | C | 3.25 | 0.43 | 0.27 | 0.70 |
| rs1031143 | 22405237 | 5 | CDH12 | A | T | 3.55 | 1.15 | 1.07 | 1.25 |
| rs931555 | 35839334 | 5 | IL7R | C | T | 6.41 | 1.25 | 1.15 | 1.36 |
| rs6896969 | 40460183 | 5 | PTGER4 | C | A | 5.28 | 1.20 | 1.11 | 1.30 |
| rs4410646 | 42605399 | 5 | GHR | A | C | 3.88 | 1.25 | 1.09 | 1.43 |
| rs10473306 | 43323194 | 5 | HMGCS1 | C | G | 3.43 | 0.61 | 0.47 | 0.80 |
| rs786334 | 52948111 | 5 | NDUFS4 | C | G | 3.55 | 0.83 | 0.75 | 0.92 |
| rs3815915 | 54288527 | 5 | ESM1 | T | A | 4.34 | 0.86 | 0.80 | 0.93 |
| rs484573 | 72476863 | 5 | TMEM171 | C | A | 3.41 | 0.81 | 0.72 | 0.92 |
| rs6892080 | 84023778 | 5 | EDIL3 | A | G | 4.13 | 1.20 | 1.09 | 1.32 |
| rs7710308 | 85465217 | 5 | COX7C | G | A | 4.88 | 1.19 | 1.10 | 1.29 |
| rs17427993 | 109423883 | 5 | MAN2A1 | T | G | 4.04 | 1.26 | 1.12 | 1.43 |
| rs1384281 | 113176773 | 5 | YTHDC2 | G | A | 3.34 | 0.85 | 0.77 | 0.93 |
| rs17427447 | 119946240 | 5 | PRR16 | A | C | 4.18 | 0.83 | 0.76 | 0.91 |
| rs329304 | 133925728 | 5 | PHF15 | T | C | 3.58 | 0.86 | 0.80 | 0.93 |
| rs1432727 | 155383883 | 5 | SGCD | A | G | 4.03 | 0.79 | 0.69 | 0.90 |
| rs2546890 | 158692478 | 5 | IL12B | G | A | 4.23 | 0.85 | 0.78 | 0.92 |
| rs10051942 | 174043508 | 5 | MSX2 | G | C | 4.85 | 1.24 | 1.13 | 1.37 |
| rs11740771 | 175879550 | 5 | RNF44 | G | T | 3.39 | 1.16 | 1.07 | 1.25 |
| rs11749438 | 178063851 | 5 | ZNF354A | C | T | 4.30 | 0.83 | 0.76 | 0.91 |
| rs12206548 | 451231 | 6 | EXOC2 | G | A | 3.66 | 0.86 | 0.79 | 0.93 |
| rs7755154 | 2558943 | 6 | C6orf195 | C | T | 4.61 | 1.21 | 1.11 | 1.32 |
| rs2765366 | 6931616 | 6 | RREB1 | G | A | 4.28 | 0.69 | 0.57 | 0.83 |
| rs11755663 | 13260894 | 6 | PHACTR1 | T | C | 5.35 | 0.80 | 0.72 | 0.88 |
| rs17419851 | 24857392 | 6 | C6orf32 | C | T | 3.33 | 0.79 | 0.69 | 0.90 |
| rs7772297 | 31436805 | 6 | HLA-B | C | G | 11.06 | 1.40 | 1.26 | 1.56 |
| rs204877 | 32155414 | 6 | TNXB | C | G | 3.09 | 0.78 | 0.68 | 0.90 |
| rs3135388 | 32521029 | 6 | DRB1 | G | A | 145.00 | 0.47 | 0.38 | 0.58 |
| rs9268148 | 32367505 | 6 | C6orf10 | A | G | 100.00 | 0.58 | 0.50 | 0.67 |
| rs9275596 | 32789609 | 6 | HLA-DQA2 | T | C | 58.11 | 0.76 | 0.69 | 0.84 |
| rs1800454 | 32908390 | 6 | TAP2 | C | T | 8.47 | 1.37 | 1.20 | 1.56 |
| rs9277565 | 33164875 | 6 | HLA-DPB1 | T | C | 5.57 | 1.25 | 1.14 | 1.38 |
| rs17578851 | 52117669 | 6 | IL17 | C | T | 3.66 | 1.23 | 1.10 | 1.37 |
| rs9361086 | 77354622 | 6 | IMPG1 | C | G | 5.59 | 1.46 | 1.25 | 1.71 |
| rs10498930 | 83423071 | 6 | UBE2CBP | T | C | 3.69 | 1.37 | 1.16 | 1.61 |
| rs2841309 | 100858611 | 6 | SIM1 | C | T | 4.85 | 0.84 | 0.78 | 0.91 |
| rs4341027 | 128348811 | 6 | PTPRK | T | G | 3.96 | 1.19 | 1.09 | 1.30 |
| rs12664247 | 129997020 | 6 | ARHGAP18 | T | C | 4.48 | 1.19 | 1.09 | 1.29 |
| rs1415701 | 130387528 | 6 | L3MBTL3 | G | A | 3.46 | 0.85 | 0.78 | 0.93 |
| rs595985 | 137968020 | 6 | TNFAIP3 | T | C | 5.12 | 1.24 | 1.13 | 1.37 |
| rs2473517 | 139470451 | 6 | HECA | C | T | 3.23 | 1.19 | 1.08 | 1.32 |
| rs9403105 | 140045546 | 6 | CITED2 | G | A | 4.62 | 1.19 | 1.10 | 1.29 |
| rs7758756 | 150979885 | 6 | PLEKHG1 | T | C | 4.42 | 1.25 | 1.13 | 1.40 |
| rs9371331 | 154595985 | 6 | PIP3-E | G | A | 4.27 | 0.81 | 0.73 | 0.91 |
| rs4708970 | 162996789 | 6 | PARK2 | T | C | 4.85 | 0.79 | 0.71 | 0.88 |
| rs11759376 | 166578652 | 6 | T | G | T | 3.48 | 0.86 | 0.79 | 0.94 |
| rs6463156 | 4703519 | 7 | MMD2 | A | G | 4.79 | 1.22 | 1.12 | 1.34 |
| rs12536822 | 11969631 | 7 | TMEM106B | A | G | 3.90 | 1.18 | 1.08 | 1.29 |
| rs17280766 | 13410609 | 7 | ETV1 | A | G | 4.37 | 1.54 | 1.25 | 1.91 |
| rs6973638 | 15170510 | 7 | TMEM195 | T | C | 3.76 | 0.87 | 0.81 | 0.94 |
| rs2214935 | 22068600 | 7 | RAPGEF5 | G | A | 3.76 | 1.18 | 1.08 | 1.29 |
| rs11769998 | 25244176 | 7 | NPVF | T | C | 4.79 | 0.70 | 0.59 | 0.82 |
| rs10951575 | 38601880 | 7 | VPS41 | A | T | 4.56 | 0.43 | 0.29 | 0.64 |
| rs6947780 | 39709259 | 7 | CDC2L5 | A | G | 3.60 | 1.20 | 1.09 | 1.34 |
| rs528733 | 56957145 | 7 | ZNF479 | A | G | 4.08 | 2.16 | 1.44 | 3.24 |
| rs4718603 | 66552866 | 7 | STAG3L4 | G | A | 3.98 | 0.87 | 0.81 | 0.94 |
| rs17869723 | 89900540 | 7 | CLDN12 | C | T | 3.74 | 0.37 | 0.22 | 0.63 |
| rs13438318 | 105855861 | 7 | PBEF1 | G | A | 4.22 | 0.55 | 0.41 | 0.73 |
| rs11771064 | 107331571 | 7 | LAMB4 | C | T | 3.61 | 2.82 | 1.62 | 4.92 |
| rs17864129 | 126336812 | 7 | GRM8 | C | T | 3.96 | 1.23 | 1.10 | 1.39 |
| rs357382 | 137462926 | 7 | AKR1D1 | G | A | 3.91 | 0.86 | 0.79 | 0.93 |
| rs10271373 | 138187050 | 7 | ZC3HAV1 | C | A | 4.69 | 0.87 | 0.80 | 0.94 |
| rs2058448 | 149884468 | 7 | GIMAP5 | C | T | 3.46 | 1.17 | 1.07 | 1.28 |
| rs10503363 | 6697003 | 8 | DEFB1 | G | C | 3.51 | 0.52 | 0.37 | 0.75 |
| rs13263210 | 10795090 | 8 | XKR6 | C | T | 3.93 | 0.72 | 0.60 | 0.87 |
| rs7839808 | 13952317 | 8 | SGCZ | A | G | 3.27 | 1.39 | 1.13 | 1.70 |
| rs8190867 | 18125521 | 8 | NAT1 | G | A | 3.32 | 0.62 | 0.47 | 0.81 |
| rs7814564 | 26835382 | 8 | ADRA1A | A | T | 3.70 | 1.38 | 1.16 | 1.65 |
| rs4739346 | 37546358 | 8 | ZNF703 | A | C | 4.46 | 0.70 | 0.59 | 0.83 |
| rs6472723 | 50897681 | 8 | SNTG1 | G | T | 3.76 | 0.35 | 0.20 | 0.61 |
| rs17395190 | 66743612 | 8 | MTFR1 | T | A | 3.93 | 1.40 | 1.18 | 1.66 |
| rs16938181 | 73431218 | 8 | LOC441353 | C | A | 3.50 | 0.55 | 0.40 | 0.76 |
| rs17249487 | 75223904 | 8 | JPH1 | A | G | 4.56 | 1.37 | 1.16 | 1.61 |
| rs2167730 | 78265972 | 8 | PXMP3 | T | C | 3.85 | 0.85 | 0.79 | 0.93 |
| rs11987948 | 88143783 | 8 | CNBD1 | A | G | 3.01 | 0.53 | 0.37 | 0.78 |
| rs16892784 | 90284765 | 8 | TRPA1 | T | C | 4.44 | 1.64 | 1.30 | 2.08 |
| rs13260237 | 105917233 | 8 | LRP12 | T | C | 4.11 | 0.83 | 0.76 | 0.91 |
| rs13251655 | 119829740 | 8 | SAMD12 | C | T | 3.45 | 1.16 | 1.07 | 1.27 |
| rs7823843 | 130464493 | 8 | CCDC26 | C | T | 4.61 | 0.52 | 0.37 | 0.74 |
| rs6577993 | 140196705 | 8 | COL22A1 | T | C | 3.32 | 1.14 | 1.05 | 1.24 |
| rs10110999 | 141441526 | 8 | CHRAC1 | G | A | 3.72 | 0.83 | 0.75 | 0.91 |
| rs618810 | 232529 | 9 | DOCK8 | A | T | 4.15 | 0.85 | 0.78 | 0.92 |
| rs10974772 | 4757677 | 9 | AK3L1 | C | T | 4.15 | 1.24 | 1.12 | 1.38 |
| rs10758669 | 4971602 | 9 | JAK2 | A | C | 3.23 | 0.86 | 0.79 | 0.94 |
| rs2918182 | 6654282 | 9 | GLDC | G | A | 3.91 | 0.88 | 0.81 | 0.95 |
| rs1339170 | 23055000 | 9 | LOC646611 | A | T | 3.77 | 0.62 | 0.48 | 0.79 |
| rs879284 | 35819031 | 9 | TMEM8B | G | A | 3.27 | 0.84 | 0.76 | 0.93 |
| rs10125429 | 68523582 | 9 | PIP5K1B | C | T | 4.23 | 2.52 | 1.61 | 3.97 |
| rs11140860 | 69753468 | 9 | C9orf135 | C | A | 4.56 | 0.76 | 0.67 | 0.87 |
| rs7026762 | 76178663 | 9 | RFK | C | T | 3.75 | 0.82 | 0.74 | 0.91 |
| rs17321848 | 83435897 | 9 | FRMD3 | T | G | 3.50 | 1.28 | 1.12 | 1.46 |
| rs935466 | 85347791 | 9 | AGTPBP1 | A | G | 3.83 | 0.85 | 0.79 | 0.93 |
| rs2492853 | 88061478 | 9 | ISCA1 | T | C | 4.93 | 0.84 | 0.76 | 0.92 |
| rs7021087 | 102409744 | 9 | CYLC2 | C | T | 3.32 | 0.67 | 0.54 | 0.84 |
| rs10124123 | 108313623 | 9 | ACTL7B | C | G | 3.38 | 0.85 | 0.77 | 0.93 |
| rs11794874 | 114835295 | 9 | TNC | C | T | 4.15 | 2.29 | 1.52 | 3.45 |
| rs1611120 | 133536855 | 9 | DBH | G | A | 4.03 | 0.65 | 0.51 | 0.81 |
| rs4401948 | 134816772 | 9 | COL5A1 | C | G | 3.20 | 1.25 | 1.10 | 1.43 |
| rs12049718 | 3907693 | 10 | KLF6 | C | A | 3.46 | 0.85 | 0.78 | 0.93 |
| rs1243982 | 8207060 | 10 | GATA3 | A | G | 4.55 | 0.81 | 0.73 | 0.89 |
| rs12268703 | 13144894 | 10 | OPTN | A | G | 4.53 | 0.83 | 0.77 | 0.91 |
| rs7080012 | 61144262 | 10 | SLC16A9 | T | A | 3.37 | 0.64 | 0.49 | 0.83 |
| rs12767186 | 61680529 | 10 | ANK3 | A | G | 4.55 | 0.82 | 0.74 | 0.90 |
| rs10822550 | 66863143 | 10 | CTNNA3 | G | A | 4.56 | 1.18 | 1.09 | 1.28 |
| rs7085781 | 77108376 | 10 | MIRN606 | C | T | 3.59 | 0.62 | 0.48 | 0.80 |
| rs3781195 | 89632589 | 10 | PTEN | A | G | 3.43 | 1.51 | 1.17 | 1.96 |
| rs304493 | 91132566 | 10 | IFIT1L | A | G | 3.42 | 0.84 | 0.77 | 0.93 |
| rs4128214 | 94538009 | 10 | EXOC6 | G | A | 3.36 | 1.16 | 1.07 | 1.26 |
| rs788086 | 95168236 | 10 | FER1L3 | C | T | 3.86 | 1.20 | 1.09 | 1.32 |
| rs479505 | 97350709 | 10 | ALDH18A1 | G | A | 4.11 | 1.26 | 1.12 | 1.42 |
| rs725076 | 105571255 | 10 | SH3PXD2A | T | G | 3.27 | 1.17 | 1.06 | 1.30 |
| rs7088797 | 110347795 | 10 | XPNPEP1 | A | T | 3.71 | 0.49 | 0.34 | 0.72 |
| rs10885868 | 117912102 | 10 | GFRA1 | T | C | 5.26 | 1.20 | 1.11 | 1.30 |
| rs2912787 | 123315528 | 10 | FGFR2 | T | C | 3.06 | 0.83 | 0.74 | 0.92 |
| rs11248608 | 125385622 | 10 | GPR26 | A | G | 3.66 | 1.38 | 1.16 | 1.64 |
| rs12260682 | 126644390 | 10 | CTBP2 | C | G | 3.40 | 0.60 | 0.44 | 0.83 |
| rs11017740 | 132816398 | 10 | TCERG1L | G | A | 3.25 | 0.64 | 0.48 | 0.86 |
| rs231899 | 2724543 | 11 | KCNQ1 | A | G | 3.06 | 1.19 | 1.07 | 1.32 |
| rs7124586 | 4702134 | 11 | OR51C4P | A | G | 3.26 | 1.21 | 1.07 | 1.37 |
| rs4758310 | 8165969 | 11 | RIC3 | A | G | 3.21 | 0.87 | 0.80 | 0.95 |
| rs7123257 | 13268830 | 11 | ARNTL | C | T | 4.24 | 0.64 | 0.52 | 0.80 |
| rs2729880 | 19449896 | 11 | E2F8 | C | T | 4.34 | 0.79 | 0.70 | 0.88 |
| rs12223355 | 25991701 | 11 | TMEM16C | G | A | 3.33 | 0.62 | 0.48 | 0.81 |
| rs11030875 | 29902283 | 11 | KCNA4 | G | A | 4.07 | 0.86 | 0.78 | 0.94 |
| rs10838251 | 44268544 | 11 | ALX4 | A | C | 3.04 | 1.17 | 1.07 | 1.29 |
| rs4939490 | 60550227 | 11 | CD6 | G | C | 9.00 | 1.30 | 1.19 | 1.42 |
| rs4576852 | 68919826 | 11 | MYEOV | T | G | 3.69 | 1.17 | 1.06 | 1.28 |
| rs6592632 | 75794014 | 11 | PRKRIR | C | T | 3.93 | 1.21 | 1.10 | 1.34 |
| rs147578 | 87267304 | 11 | RAB38 | A | G | 3.08 | 0.86 | 0.79 | 0.94 |
| rs655763 | 108682027 | 11 | C11orf87 | C | T | 6.03 | 1.59 | 1.32 | 1.92 |
| rs4936323 | 114596329 | 11 | CADM1 | T | G | 3.40 | 1.18 | 1.07 | 1.29 |
| rs4938573 | 118247052 | 11 | BLR1 | T | C | 4.37 | 1.24 | 1.12 | 1.37 |
| rs10892613 | 120063294 | 11 | GRIK4 | G | A | 3.14 | 0.83 | 0.74 | 0.92 |
| rs12419184 | 125561518 | 11 | RPUSD4 | C | T | 6.03 | 0.72 | 0.63 | 0.82 |
| rs541969 | 125965209 | 11 | KIRREL3 | C | T | 4.01 | 0.85 | 0.79 | 0.92 |
| rs10893663 | 126660197 | 11 | PRR10 | G | A | 3.10 | 1.29 | 1.08 | 1.54 |
| rs10894287 | 130313693 | 11 | SNX19 | T | C | 3.97 | 0.86 | 0.79 | 0.93 |
| rs7928505 | 132794724 | 11 | OPCML | G | C | 3.74 | 2.81 | 1.64 | 4.83 |
| rs1800693 | 6310270 | 12 | TNFRSF1A | C | T | 3.89 | 1.20 | 1.10 | 1.32 |
| rs12300846 | 9634751 | 12 | KLRB1 | T | A | 4.48 | 0.84 | 0.77 | 0.91 |
| rs220584 | 13852165 | 12 | GRIN2B | G | A | 4.35 | 0.59 | 0.46 | 0.76 |
| rs12581372 | 24232855 | 12 | SOX5 | C | T | 4.44 | 1.34 | 1.14 | 1.58 |
| rs1382856 | 25628257 | 12 | IFLTD1 | G | A | 3.59 | 0.78 | 0.68 | 0.89 |
| rs12426074 | 26534577 | 12 | ITPR2 | A | T | 3.92 | 0.83 | 0.75 | 0.91 |
| rs7314705 | 30090640 | 12 | TMTC1 | T | C | 4.63 | 1.53 | 1.26 | 1.86 |
| rs17625294 | 39808407 | 12 | CNTN1 | G | A | 3.33 | 1.21 | 1.09 | 1.35 |
| rs1452106 | 41220974 | 12 | PRICKLE1 | A | G | 3.28 | 0.86 | 0.79 | 0.94 |
| rs2407121 | 41598192 | 12 | MRPS36P5 | A | T | 3.77 | 0.78 | 0.69 | 0.89 |
| rs12368653 | 56419523 | 12 | CENTG1 | G | A | 4.34 | 0.85 | 0.79 | 0.92 |
| rs1436665 | 57633331 | 12 | LRIG3 | G | A | 5.54 | 0.77 | 0.69 | 0.86 |
| rs11173686 | 59666335 | 12 | FAM19A2 | T | C | 3.89 | 0.83 | 0.75 | 0.91 |
| rs1908666 | 67661472 | 12 | CPM | C | G | 3.80 | 1.16 | 1.07 | 1.25 |
| rs7296395 | 79087750 | 12 | C12orf64 | C | T | 5.43 | 0.60 | 0.48 | 0.76 |
| rs10862520 | 81795565 | 12 | TMTC2 | A | G | 4.10 | 0.83 | 0.76 | 0.92 |
| rs7960353 | 83528404 | 12 | SLC6A15 | A | G | 4.09 | 0.83 | 0.75 | 0.91 |
| rs3924690 | 92026763 | 12 | EEA1 | C | G | 3.40 | 1.24 | 1.10 | 1.40 |
| rs1558802 | 107538714 | 12 | SELPLG | T | A | 3.54 | 0.84 | 0.77 | 0.92 |
| rs11065987 | 110535144 | 12 | BRAP | A | G | 5.40 | 0.83 | 0.76 | 0.90 |
| rs1104831 | 116387282 | 12 | KSR2 | G | A | 4.15 | 0.84 | 0.77 | 0.91 |
| rs10431386 | 119591646 | 12 | KIAA0152 | C | T | 3.91 | 0.85 | 0.78 | 0.92 |
| rs837466 | 123535327 | 12 | NCOR2 | T | C | 3.33 | 0.84 | 0.76 | 0.93 |
| rs768516 | 126639482 | 12 | SLC15A4 | C | T | 3.18 | 1.97 | 1.33 | 2.92 |
| rs9553989 | 26783488 | 13 | RASL11A | A | G | 3.38 | 0.83 | 0.75 | 0.92 |
| rs927544 | 46354052 | 13 | HTR2A | A | G | 5.06 | 1.22 | 1.12 | 1.33 |
| rs9526480 | 48052578 | 13 | RCBTB2 | A | G | 4.34 | 0.55 | 0.41 | 0.73 |
| rs9596270 | 49740441 | 13 | DLEU1 | T | C | 7.08 | 1.56 | 1.31 | 1.85 |
| rs9535449 | 49962302 | 13 | DLEU7 | T | C | 4.76 | 0.74 | 0.65 | 0.85 |
| rs17482764 | 51628260 | 13 | NEK3 | T | A | 3.49 | 1.88 | 1.33 | 2.65 |
| rs9542997 | 72024083 | 13 | DACH1 | T | C | 3.09 | 0.85 | 0.77 | 0.93 |
| rs17067629 | 76846698 | 13 | MYCBP2 | T | C | 3.53 | 0.69 | 0.57 | 0.85 |
| rs1538337 | 94856458 | 13 | CLDN10 | T | A | 4.18 | 1.18 | 1.09 | 1.27 |
| rs7337143 | 102624684 | 13 | SLC10A2 | A | G | 3.88 | 0.43 | 0.28 | 0.67 |
| rs1642686 | 105047651 | 13 | DAOA | C | A | 3.48 | 0.87 | 0.80 | 0.94 |
| rs10146906 | 23603379 | 14 | CPNE6 | C | A | 5.60 | 1.30 | 1.16 | 1.45 |
| rs225855 | 29657983 | 14 | PRKD1 | G | A | 3.30 | 1.19 | 1.08 | 1.32 |
| rs1951223 | 38458106 | 14 | SEC23A | G | C | 4.22 | 0.84 | 0.77 | 0.91 |
| rs8010450 | 46799461 | 14 | RPL13AP2 | A | G | 3.91 | 0.85 | 0.78 | 0.92 |
| rs17721685 | 56781699 | 14 | SEC10L1 | T | A | 3.65 | 1.54 | 1.22 | 1.93 |
| rs10148925 | 59157663 | 14 | RTN1 | A | G | 3.23 | 1.34 | 1.09 | 1.64 |
| rs1420810 | 86572905 | 14 | LOC283585 | G | A | 4.11 | 0.63 | 0.50 | 0.80 |
| rs12897048 | 96715934 | 14 | VRK1 | C | T | 4.00 | 1.25 | 1.12 | 1.40 |
| rs7173839 | 24219029 | 15 | GABRB3 | G | C | 4.06 | 0.38 | 0.23 | 0.62 |
| rs1961021 | 31350818 | 15 | RYR3 | C | T | 4.10 | 1.19 | 1.08 | 1.31 |
| rs16967121 | 36710299 | 15 | C15orf53 | A | G | 3.72 | 1.32 | 1.12 | 1.56 |
| rs199138 | 43174842 | 15 | DUOX2 | G | A | 4.63 | 1.83 | 1.38 | 2.41 |
| rs3098171 | 48558803 | 15 | USP8 | G | C | 4.89 | 1.19 | 1.10 | 1.29 |
| rs17304143 | 60364343 | 15 | FLJ38723 | G | T | 4.00 | 0.80 | 0.72 | 0.90 |
| rs4932507 | 89976286 | 15 | TRNAY16P | G | A | 4.86 | 0.83 | 0.76 | 0.90 |
| rs4777989 | 90807744 | 15 | ST8SIA2 | A | G | 3.55 | 0.87 | 0.80 | 0.94 |
| rs1424699 | 92943172 | 15 | MCTP2 | A | G | 3.71 | 0.34 | 0.19 | 0.61 |
| rs11073379 | 93029048 | 15 | LOC440311 | A | G | 3.47 | 0.82 | 0.73 | 0.92 |
| rs9940149 | 240642 | 16 | ITFG3 | G | A | 3.50 | 1.17 | 1.05 | 1.29 |
| rs415457 | 1001059 | 16 | SOX8 | G | C | 3.86 | 0.75 | 0.65 | 0.87 |
| rs11077338 | 3444786 | 16 | FLJ14154 | A | G | 3.10 | 0.75 | 0.63 | 0.89 |
| rs11076884 | 5134491 | 16 | FAM86A | G | C | 3.75 | 1.30 | 1.12 | 1.51 |
| rs10852680 | 7215399 | 16 | A2BP1 | C | T | 3.75 | 0.85 | 0.78 | 0.93 |
| rs7187161 | 12602404 | 16 | SNX29 | C | T | 4.70 | 0.85 | 0.78 | 0.91 |
| rs11863274 | 22957125 | 16 | USP31 | A | G | 4.05 | 0.66 | 0.54 | 0.81 |
| rs17260689 | 53104314 | 16 | IRX3 | C | T | 3.78 | 0.80 | 0.71 | 0.90 |
| rs1585145 | 54442124 | 16 | CES1 | A | G | 3.85 | 0.54 | 0.39 | 0.74 |
| rs10775336 | 56103237 | 16 | CCDC102A | C | G | 4.51 | 0.72 | 0.62 | 0.85 |
| rs1050779 | 56637716 | 16 | MMP15 | C | G | 4.86 | 0.80 | 0.72 | 0.88 |
| rs9931083 | 77856003 | 16 | WWOX | C | T | 5.54 | 0.54 | 0.42 | 0.70 |
| rs3751804 | 85103728 | 16 | FOXF1 | T | C | 3.32 | 0.78 | 0.67 | 0.90 |
| rs9924445 | 85304319 | 16 | FOXL1 | G | A | 4.87 | 0.38 | 0.25 | 0.59 |
| rs16966539 | 87084993 | 16 | ZFPM1 | A | G | 3.72 | 2.30 | 1.48 | 3.56 |
| rs8066941 | 9529175 | 17 | USP43 | T | G | 3.58 | 0.85 | 0.77 | 0.93 |
| rs7208260 | 9683463 | 17 | GLP2R | C | G | 3.32 | 0.74 | 0.61 | 0.89 |
| rs1024367 | 9995901 | 17 | GAS7 | T | C | 4.12 | 0.82 | 0.75 | 0.91 |
| rs4985700 | 16806800 | 17 | TNFRSF13B | A | C | 4.16 | 1.20 | 1.09 | 1.32 |
| rs11658169 | 19812514 | 17 | AKAP10 | C | T | 3.99 | 0.83 | 0.76 | 0.91 |
| rs280046 | 29006254 | 17 | ACCN1 | A | T | 3.86 | 0.85 | 0.78 | 0.92 |
| rs2097761 | 29511447 | 17 | CCL2 | A | G | 3.04 | 0.81 | 0.72 | 0.92 |
| rs2304494 | 37216477 | 17 | SC65 | C | T | 4.61 | 1.49 | 1.24 | 1.79 |
| rs9901869 | 42930205 | 17 | NPEPPS | A | G | 4.94 | 0.84 | 0.78 | 0.91 |
| rs8078184 | 46020143 | 17 | CACNA1G | A | G | 4.78 | 1.20 | 1.11 | 1.31 |
| rs7220996 | 62235162 | 17 | PRKCA | G | A | 4.52 | 0.58 | 0.45 | 0.75 |
| rs11870121 | 71370533 | 17 | WBP2 | A | G | 4.99 | 0.80 | 0.73 | 0.89 |
| rs661059 | 7678140 | 18 | PTPRM | A | G | 4.01 | 0.83 | 0.76 | 0.91 |
| rs8091169 | 8827440 | 18 | KIAA0802 | G | A | 3.23 | 1.14 | 1.05 | 1.23 |
| rs7240134 | 13822160 | 18 | MC5R | G | A | 4.07 | 0.77 | 0.68 | 0.88 |
| rs7242930 | 20461288 | 18 | RAC1P1 | T | C | 4.00 | 1.16 | 1.07 | 1.26 |
| rs528438 | 23927873 | 18 | CDH2 | T | C | 5.56 | 0.78 | 0.70 | 0.86 |
| rs9965182 | 53928699 | 18 | NEDD4L | C | T | 3.70 | 0.79 | 0.68 | 0.90 |
| rs8083624 | 54385169 | 18 | ALPK2 | T | A | 3.86 | 1.30 | 1.14 | 1.49 |
| rs12954376 | 57035772 | 18 | CDH20 | T | A | 5.54 | 0.82 | 0.75 | 0.89 |
| rs1016860 | 58946054 | 18 | BCL2 | C | T | 3.69 | 0.78 | 0.68 | 0.89 |
| rs426921 | 63402133 | 18 | DSEL | C | T | 5.89 | 1.28 | 1.14 | 1.43 |
| rs7237611 | 65700586 | 18 | CD226 | A | G | 3.46 | 0.86 | 0.79 | 0.93 |
| rs12604138 | 67513820 | 18 | LOC643765 | A | G | 3.38 | 1.38 | 1.14 | 1.66 |
| rs1273210 | 70224704 | 18 | C18orf51 | G | A | 3.62 | 0.35 | 0.19 | 0.64 |
| rs1867205 | 70957438 | 18 | ZNF407 | T | C | 3.27 | 2.92 | 1.59 | 5.34 |
| rs7506886 | 74152382 | 18 | LOC400662 | C | T | 3.43 | 1.45 | 1.18 | 1.77 |
| rs7250872 | 1762603 | 19 | ATP8B3 | T | C | 4.07 | 0.83 | 0.74 | 0.91 |
| rs1862511 | 6537268 | 19 | CD70 | G | A | 3.61 | 0.78 | 0.68 | 0.89 |
| rs7249323 | 13238865 | 19 | CACNA1A | A | G | 4.18 | 0.82 | 0.75 | 0.90 |
| rs1229934 | 21867070 | 19 | ZNF43 | G | A | 4.60 | 0.32 | 0.18 | 0.59 |
| rs10418434 | 46959704 | 19 | CEACAM6 | T | C | 3.82 | 1.17 | 1.08 | 1.27 |
| rs2288481 | 54570008 | 19 | DKKL1 | G | A | 4.55 | 0.82 | 0.74 | 0.90 |
| rs12609896 | 62291700 | 19 | USP29 | G | A | 3.20 | 0.83 | 0.75 | 0.92 |
| rs1569683 | 12016758 | 20 | BTBD3 | G | A | 3.65 | 0.81 | 0.72 | 0.90 |
| rs6043703 | 15988774 | 20 | MACROD2 | C | T | 3.31 | 1.25 | 1.10 | 1.42 |
| rs6044386 | 16750429 | 20 | OTOR | A | G | 3.73 | 0.84 | 0.77 | 0.92 |
| rs6131888 | 16925054 | 20 | PCSK2 | A | G | 3.13 | 0.87 | 0.80 | 0.94 |
| rs4813468 | 22727092 | 20 | KRT18P3 | T | A | 3.51 | 1.21 | 1.09 | 1.36 |
| rs878279 | 40042857 | 20 | PTPRT | A | G | 3.40 | 1.16 | 1.07 | 1.26 |
| rs932905 | 48229538 | 20 | CEBPB | G | A | 4.62 | 1.22 | 1.11 | 1.33 |
| rs2133618 | 53053218 | 20 | DOK5 | T | C | 3.03 | 0.69 | 0.56 | 0.86 |
| rs6064542 | 55394763 | 20 | RAE1 | C | T | 3.66 | 0.64 | 0.50 | 0.82 |
| rs6128290 | 56222652 | 20 | C20orf86 | T | C | 3.30 | 1.28 | 1.11 | 1.47 |
| rs2824323 | 17757662 | 21 | BTG3 | A | T | 3.61 | 0.73 | 0.62 | 0.86 |
| rs219714 | 26673337 | 21 | CYYR1 | T | A | 3.87 | 0.80 | 0.71 | 0.90 |
| rs845960 | 31621988 | 21 | TIAM1 | A | G | 3.92 | 1.22 | 1.10 | 1.35 |
| rs2839578 | 43025706 | 21 | PDE9A | G | C | 4.45 | 1.18 | 1.09 | 1.29 |
| rs11704699 | 16144735 | 22 | CECR1 | T | G | 3.09 | 0.81 | 0.72 | 0.92 |
| rs1640350 | 18584917 | 22 | RTN4R | T | C | 3.75 | 1.21 | 1.09 | 1.33 |
| rs741195 | 19318320 | 22 | PCQAP | G | C | 3.49 | 1.18 | 1.08 | 1.30 |
| rs11703395 | 21351763 | 22 | GGTL4 | G | A | 3.26 | 1.64 | 1.24 | 2.18 |
| rs4447 | 31599694 | 22 | SYN3 | T | C | 6.10 | 0.74 | 0.66 | 0.83 |
| rs3788551 | 38077027 | 22 | SYNGR1 | A | T | 3.08 | 0.77 | 0.66 | 0.90 |
| rs135382 | 47118481 | 22 | FAM19A5 | C | T | 4.27 | 1.28 | 1.14 | 1.45 |
